# Compact single-shot metalens depth sensors inspired by eyes of jumping spiders

**DOI:** 10.1073/pnas.1912154116

**Published:** 2019-10-28

**Authors:** Qi Guo, Zhujun Shi, Yao-Wei Huang, Emma Alexander, Cheng-Wei Qiu, Federico Capasso, Todd Zickler

**Affiliations:** ^a^John A. Paulson School of Engineering and Applied Sciences, Harvard University, Cambridge, MA 02138;; ^b^Department of Physics, Harvard University, Cambridge, MA 02138;; ^c^Department of Electrical and Computer Engineering, National University of Singapore, 117580 Singapore;; ^d^Department of Electrical Engineering and Computer Science, University of California, Berkeley, CA 94720

**Keywords:** depth sensor, metalens, jumping spider

## Abstract

Jumping spiders (Salticidae) rely on accurate depth perception for predation and navigation. They accomplish depth perception, despite their tiny brains, by using specialized optics. Each principal eye includes a multitiered retina that simultaneously receives multiple images with different amounts of defocus, and from these images, distance is decoded with relatively little computation. We introduce a compact depth sensor that is inspired by the jumping spider. It combines metalens optics, which modifies the phase of incident light at a subwavelength scale, with efficient computations to measure depth from image defocus. Instead of using a multitiered retina to transduce multiple simultaneous images, the sensor uses a metalens to split the light that passes through an aperture and concurrently form 2 differently defocused images at distinct regions of a single planar photosensor. We demonstrate a system that deploys a 3-mm-diameter metalens to measure depth over a 10-cm distance range, using fewer than 700 floating point operations per output pixel. Compared with previous passive depth sensors, our metalens depth sensor is compact, single-shot, and requires a small amount of computation. This integration of nanophotonics and efficient computation brings artificial depth sensing closer to being feasible on millimeter-scale, microwatts platforms such as microrobots and microsensor networks.

## Significance

Nature provides diverse solutions to passive visual depth sensing. Evolution has produced vision systems that are highly specialized and efficient, delivering depth-perception capabilities that often surpass those of existing artificial depth sensors. Here, we learn from the eyes of jumping spiders and demonstrate a metalens depth sensor that shares the compactness and high computational efficiency of its biological counterpart. Our device combines multifunctional metalenses, ultrathin nanophotonic components that control light at a subwavelength scale, and efficient computations to measure depth from image defocus. Compared with previous passive artificial depth sensors, our bioinspired design is lightweight, single-shot, and requires a small amount of computation. The integration of nanophotonics and efficient computation establishes a paradigm for design in computational sensing.

Visual depth sensors combine cameras, computational algorithms, and sometimes light sources to sense the 3-dimensional shapes of surrounding objects and scenes. Lidar systems (1), time-of-flight cameras (2–8), and structured lighting systems (9, 10) are examples of depth sensors that use active light sources, whereas binocular stereo systems (11) and light-field cameras (12–14) are examples that are passive, relying solely on the ambient light that happens to be available. These approaches have found widespread use on autonomous vehicles, drones, mobile phones, and many other platforms. However, they require either active lighting or iterative computation and optimization, and are thus not well suited to low-power platforms, such as mobile sensor networks and robotic insects (15–17), which impose much more severe constraints on size, weight, and power consumption.

Alternative methods that utilize optical defocus to measure depth have been demonstrated to potentially greatly reduce the amount of depth computation and require no active lighting (18, 19). These algorithms (18–23) compute depth by comparing 2 differently defocused images of the same scene and produce a depth map, comprising a depth value at each pixel. However, one major challenge with this method is the optics. With conventional optical components, capturing 2 differently defocused images usually requires making physical changes to the optical system, such as reducing or enlarging its aperture (20, 21, 23) or deforming its lens (19). This not only adds significant complexity to the system control, but also fundamentally limits the depth-sensing performance by introducing unwanted delays and motion artifacts. Some previous algorithms use look-up tables (20) or iterative methods (22) in their framework to measure depth. However, these methods are hard to implement in a differentiable manner and rely on exhaustive search, instead of using gradient-based search methods, to determine the required parameters.

To address these challenges, we introduce the metalens depth sensor. It is compact, static, single-shot, and requires low computational power. Thanks to the versatile wavefront-shaping capability of metalenses, ultrathin nanophotonic components that can tailor arbitrary optical wavefront at a subwavelength scale, our device can simultaneously capture 2 differently defocused images through the same aperture without having to make physical changes to the optical system. It avoids the artifacts usually incurred by reimaging over time while changing a camera’s optics and can potentially improve the depth sensor’s time resolution. Besides, the image-processing algorithm is completely differentiable, which enables data-driven, gradient-based calibration of the computational parameters compared to the nondifferentiable methods (20, 22).

**Fig. 1.**
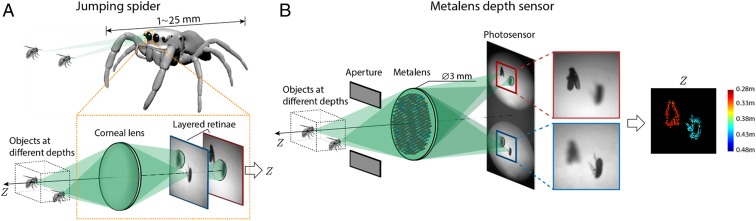
Jumping spider and metalens depth sensor. (*A*) Jumping spiders can sense depth using either 1 of their 2 front-facing principal eyes (highlighted). Unlike the single retina found in human eyes, jumping spiders have multiple retinae that are layered and semitransparent. The layered-retinae structure can simultaneously measure multiple images of the same scene with different amounts of defocus, and behavioral evidence suggests that spiders measure depth using the defocus cues that are available in these images (26). (*B*) The metalens depth sensor estimates depth by mimicking the jumping spider. It uses a metalens to simultaneously capture 2 images with different defocus, and it uses efficient calculations to produce depth from these images. The jumping spider’s depth perception operates normally under green light (26), and we similarly designed the metalens to operate at a wavelength of 532 nm. We coupled the metalens with a spectral filter to limit the spectral bandwidth and with a rectangular aperture to prevent overlap between the 2 adjacent images. The images depicted on the photosensor were taken from experiments and show 2 fruit flies located at different distances. The corresponding depth map computed by the sensor is shown on the right, with color used to represent object distance. The closer and farther flies are colored red and blue, respectively.

The working principle is inspired by the eyes of jumping spiders (Salticidae), which use defocus to succeed at sensing depth, despite the fact that their brains are about as small as poppy seeds (24). Each of the spider’s principal eyes includes a specialized structure (25) with stacked translucent retinae that simultaneously observe the world with different amounts of optical defocus (Fig. 1*A*). Behavioral experiments have shown that the input from 1 principal eye suffices for a jumping spider to sense depth accurately enough to leap onto prey from distances of several body lengths and that depth perception can be predictably manipulated by changing the ambient light spectrum in a way that distorts the optical defocus (26).

Inspired by the specialized compact optical structure of the jumping spider’s principal eye, we propose to use metasurface technology (27–34) to simultaneously collect a pair of differently defocused images using the 2 halves of a single planar photosensor (Fig. 1*B*). Metasurfaces are ultrathin planar optical components consisting of subwavelength-spaced nanostructures patterned at an interface (27). By engineering the shape of individual nanostructures, one can control the phase, amplitude, and polarization of the transmitted wavefront at subwavelength scales, allowing multiple functions to be multiplexed within a single device. Metasurfaces have enabled a variety of optical devices with capabilities that surpass those of conventional refractive or diffractive elements, ranging from high-performance imaging lenses (metalenses) (29, 35) to novel polarization holograms (36). In our prototype sensor, we encode 2 complementary lens phase profiles with distinct focal lengths and lateral offsets on a shared aperture in a single metalens by spatial multiplexing (28). In this way, 2 differently defocused images can be captured simultaneously side by side on the photosensor in a single shot. We design the metalens focal lengths together using a depth-reconstruction algorithm, so that accurate depth maps can be computed from the 2 simultaneous images with calculations that are spatially localized and few in number—i.e., depth computations for each image pixel involve only a small spatial neighborhood of pixels and require no additional correspondence search after initial calibration.

Our prototype produces depth values over a range of 10 cm from single-shot measurements using a millimeter-scale metalens. Calculating depth at each output pixel of the depth map requires fewer than 700 floating point operations (FLOPs) and involves the digitized intensity values in only a 25 × 25 spatial neighborhood of pixels. This integration of nanophotonics and efficient computation brings artificial depth sensing closer to being feasible on millimeter-scale, microwatts platforms such as microrobots and microsensor networks.

## Principle

**Fig. 2.**
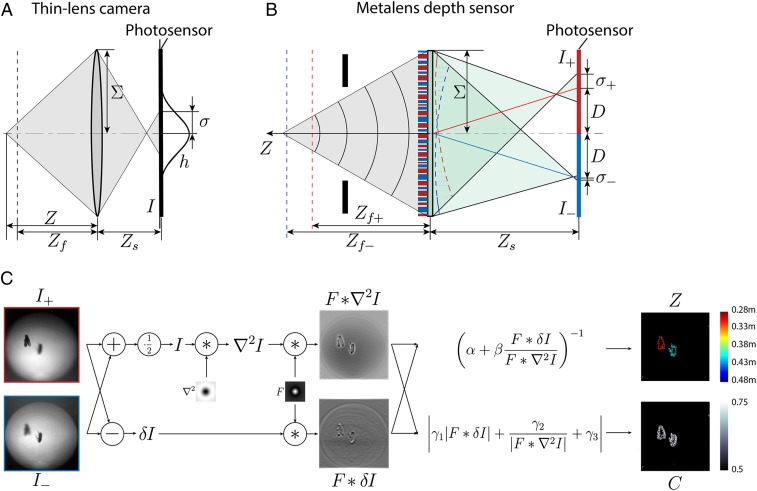
Operating principle. (*A*) A conventional thin-lens camera, in which the PSF width σ on the photosensor is determined by the optics and the depth Z (the object distance) according to the lens equation (Eq. **1**). $Z_{s}$ is the distance between the lens and the photosensor. $Z_{f}$ is the in-focus distance. Σ is the entrance pupil (lens) radius. The solid black curve next to the photosensor represents a vertical cut of the PSF h, which is drawn here with a Gaussian shape. (*B*) The metalens depth sensor encodes the phase profiles of 2 thin lenses in 1 aperture. The 2 effective lenses have distinct in-focus distances ($Z_{f_{+}},Z_{f_{-}}$) (red and blue) and off-axis alignments that create 2 adjacent images ($I_{+},I_{-}$) with different PSF widths ($\sigma_{+},\sigma_{-}$). The effective image centers are shifted from the optical axis by ±D. The dashed red and blue curves next to the metalens show the transmitted wavefronts. Due to spatial multiplexing, the overall phase profile is highly discontinuous and therefore cannot be easily achieved with conventional (Fresnel) diffractive optical elements. (*C*) From a pair of input images ($I_{+},I_{-}$), a small set of calculations was used to produce the depth at each pixel across the image, generating a depth map Z(x,y) according to Eq. **5**. A confidence map C(x,y) that indicates the precision of the depth prediction at each pixel was computed alongside, according to Eq. **6**. The computation flows from left to right, beginning with the per-pixel mean $I=\frac{1}{2}\left(I_{+}+I_{-}\right)$ and difference $\delta I=I_{+}-I_{-}$; Laplacian of the average image $\nabla^{2}I$ computed by convolving the average image with a discrete Laplacian filter; and convolution with a band-pass filter *F* to attenuate noise and vignetting. From $F\;*\;\nabla^{2}I$ and F * δI, the depth and confidence map Z and C were computed by Eqs. **5** and **6**. Parameters $\alpha ,\beta ,\gamma_{1},\gamma_{2}$, and $\gamma_{3}$ were determined by the optics and were precalibrated. To eliminate large errors in the depth map, we thresholded it by showing only pixels with confidence values greater than 0.5.

We model the image I(x,y) formed on a photosensor as the convolution of the camera point spread function (PSF) with the magnified, all-in-focus object pattern as it would be observed with a pinhole camera. The camera PSF is the image captured on the photosensor when the object is a point light source. The width of the PSF depends on the optics and the distance *Z* between the object and the lens. For an ideal thin-lens camera, depicted in Fig. 2*A*, the PSF width σ is related to object distance Z by the thin-lens equation:

$$\sigma =\left[\left(\frac{1}{Z_{f}}-\frac{1}{Z}\right)Z_{s}\right]\Sigma ,$$ [1]

where $Z_{s}$ is the distance between the lens and the photosensor, Σ is the radius of the entrance pupil, and $Z_{f}$ is the in-focus distance—i.e., the distance for which the PSF width σ is equal to zero. (*SI Appendix*, section 1.1) On the right side of Eq. **1**, all quantities but the object distance *Z* are known quantities determined by the optical system. Thus, for a calibrated camera, determining the PSF width σ is equivalent to measuring object distance Z.

The PSF width σ determines the amount of image blur. An object appears sharp when its distance Z is equal to the in-focus distance $Z_{f}$ because then the PSF width σ is zero (under ray optics approximation). Conversely, when the object distance Z deviates from $Z_{f}$, the PSF width, σ, is nonzero, and the image is blurry. Recovering the PSF width (and thus depth) from a single blurry image is ill-posed without prior information about the underlying object pattern. However, when a second image of the same scene is captured with a different amount of blur, the width σ can be determined directly from the contrast change between the 2 images. One way to understand this is to assume that the PSFs can be approximated as Gaussian functions that depend on the PSF width σ:

$$h\left(x,y\right)=\frac{1}{2\pi \sigma^{2}}\exp-\frac{x^{2}+y^{2}}{2\sigma^{2}},$$ [2]

where (x,y) is the pixel position on the photosensor. Gaussian functions have the property that partial derivatives with respect to width σ and location (x,y) satisfy

$$\frac{1}{\sigma }\frac{\partial h\left(x,y\right)}{\partial \sigma }=\left(\partial_{x}^{2}+\partial_{y}^{2}\right)h\left(x,y\right)\equiv \nabla^{2}h\left(x,y\right),$$ [3]

and because the defocused image I(x,y) is the convolution of the PSF and the all-in-focus object pattern (which does not depend on σ), the same relationship between derivatives applies to the captured image:

$$\frac{1}{\sigma }\frac{\partial I\left(x,y\right)}{\partial \sigma }=\nabla^{2}I\left(x,y\right).$$ [4]

Eq. **4** indicates that σ (and thus depth *Z* through Eq. **1**) can be determined directly from the spatial Laplacian of the image $\nabla^{2}I\left(x,y\right)$ and the differential change of intensity with respect to varying PSF width $\frac{\partial I\left(x,y\right)}{\partial \sigma }$ (18, 19). The latter can be estimated via a finite difference, i.e., $\frac{\partial I\left(x,y\right)}{\partial \sigma }\approx \frac{\delta I\left(x,y\right)}{\delta \sigma }$, where δI(x,y) is the change of image intensity induced by a small, known variation of the PSF width (δσ). According to Eq. **1**, since in general no control can be made over the object distance Z, the only way to change the PSF width σ when shooting an object is to vary the parameters of the optical system, such as the sensor distance $Z_{s}$ or the in-focus distance $Z_{f}$.

A jumping spider’s principal eye can use its transparent layered retinae to simultaneously measure the (minimally) 2 images that are required to compute the finite difference δI(x,y) (Fig. 1*A*) because these retinae effectively capture images with different sensor distances $Z_{s}$. In contrast, we design a metalens that generates 2 images ($I_{+}\left(x,y\right)$, $I_{-}\left(x,y\right)$) side by side (Fig. 1*B*), with the images being equivalent to images that are captured with different in-focus distances ($Z_{f_{+}}$, $Z_{f_{-}}$) through the same pupil. We design the in-focus distances so that the difference in blur between the images, $\delta \sigma =\sigma_{+}-\sigma_{-}=\Sigma Z_{s}\left(\frac{1}{Z_{f_{+}}}-\frac{1}{Z_{f_{-}}}\right)$, is small and approximately differential. From these 2 images (Fig. 2 *C*, *Left*), we compute the per-pixel difference $\delta I\left(x,y\right)=I_{+}\left(x,y\right)-I_{-}\left(x,y\right)$ and the image Laplacian $\nabla^{2}I\left(x,y\right)$. The latter is obtained by convolving the averaged image $I\left(x,y\right)=\frac{1}{2}\left(I_{+}\left(x,y\right)+I_{-}\left(x,y\right)\right)$ with a Laplacian filter, denoted $\nabla^{2}\left(x,y\right)$. To reduce the effects of sensor noise and optical nonidealities like vignetting, δI(x,y) and $\nabla^{2}I\left(x,y\right)$ are spatially convolved with a purposefully designed linear filter F(x,y). To the lowest order, the filter F(x,y) is similar to a Gaussian filter that averages over neighboring pixels (*SI Appendix*, sections 1.3 and 2.2). The filtered results F(x,y)*δI(x,y) and $F\left(x,y\right)*\nabla^{2}I\left(x,y\right)$ are shown in Fig. 2 *C*, *Center*. Finally, we combine Eqs. **1** and **4** to calculate the depth Z at each pixel (x,y):

$$Z\left(x,y\right)={\left(\alpha +\beta \frac{F\left(x,y\right)*\delta I\left(x,y\right)}{F\left(x,y\right)*\nabla^{2}I\left(x,y\right)}\right)}^{-1},$$ [5]

with $\alpha =\frac{1}{2}\left(\frac{1}{Z_{f_{+}}}+\frac{1}{Z_{f_{-}}}\right)$ and $\beta =-{\left(\Sigma Z_{s}\delta \sigma \right)}^{-1}$ being constants that are determined by the optics. The correctness of Eq. **5** follows from the fact that Eq. **4** still holds when values δI(x,y) and $\nabla^{2}I\left(x,y\right)$ are replaced by their filtered versions F(x,y)*δI(x,y) and $F\left(x,y\right)*\nabla^{2}I\left(x,y\right)$.

In practice, even with filtering, random noise in the captured images ($I_{+}\left(x,y\right)$, $I_{-}\left(x,y\right)$) results in errors in the measured depth Z(x,y). The error can be quantified in terms of the SD of the measured depth at each pixel, which can be approximated by the measurable quantity

$$\begin{array}{cc} s_{Z}\left(x,y\right)= & \left|\gamma_{1}|F\left(x,y\right)*\delta I\left(x,y\right)|\right.\\ & +\left.\gamma_{2}|F\left(x,y\right)*\nabla^{2}I\left(x,y\right){|}^{-1}+\gamma_{3}\right|, \end{array}$$ [6]

with constants $\gamma_{1},\gamma_{2},\gamma_{3}$ that are determined by the optics (*SI Appendix*, section 1.2). This measurable quantity $s_{Z}\left(x,y\right)$ can serve as an indicator of the reliability of the measured depth Z(x,y) at each pixel (x,y). For convenience, we normalize the values of $s_{Z}\left(x,y\right)$ to the range (0,1) and define this normalized value as the confidence C(x,y). A higher confidence value C at pixel location (x,y) indicates a smaller value of $s_{Z}$ and a more accurate depth measurement Z (*SI Appendix*, section 1.2). Physically, the confidence C(x,y) characterizes the expected accuracy of the measurement at each pixel (x,y): A larger confidence value C(x,y) at a pixel indicates a statistically smaller error in the depth measurement.

Since Eq. **5** calculates depth using simple, local calculations, it fails in regions of the images that have uniform intensity and thus no measurable contrast for δI(x,y) and $\nabla^{2}I\left(x,y\right)$. To automatically identify the locations of these failures, we use the confidence score C(x,y) as a criterion, and we report depth only at pixels (x,y) whose confidence is above a certain threshold. The choice of confidence threshold affects the depth resolution, which we define as the smallest depth difference that can be resolved within a certain confidence range. In this paper, with a confidence threshold of 0.5, we achieve a depth resolution of about 5% of the object distance over the distance range [0.3m,0.4m] (see Fig. 4*B*).

The complete sequence of calculations for the depth map and confidence map is depicted in Fig. 2*C*. For visualization, the depth map is thresholded by confidence to show only the depth at pixels where the latter is greater than 0.5.

## Metalens Design and Characterization

The metalens is designed to incorporate phase profiles of 2 off-axis lenses with different in-focus distances on a shared aperture. For each off-axis lens, the required phase profile is an offset convex shape determined by in-focus distances $\left(Z_{f_{+}},Z_{f_{-}}\right)$, sensor distance $Z_{s}$, and transverse displacement of the image center ±D (Fig. 2*B*):

$$\begin{array}{ll} \phi_{\pm }\left(x,y\right) & =-\frac{2\pi }{\lambda }\left(\sqrt{x^{2}+y^{2}+Z_{f_{\pm }}^{2}}+\sqrt{x^{2}+{\left(y\mp D\right)}^{2}+Z_{s}^{2}}\right.\\ & \;\left.-\sqrt{D^{2}+Z_{s}^{2}}-Z_{f_{\pm }}\right). \end{array}$$ [7]

Here, (x,y) indicates location on the metalens. The overall phase profile is achieved by spatially interleaving the 2, $\phi_{+}\left(x,y\right)$ and $\phi_{-}\left(x,y\right)$, on the metalens at a subwavelength scale. The design specifications are in *SI Appendix*, section 2.1.

**Fig. 3.**
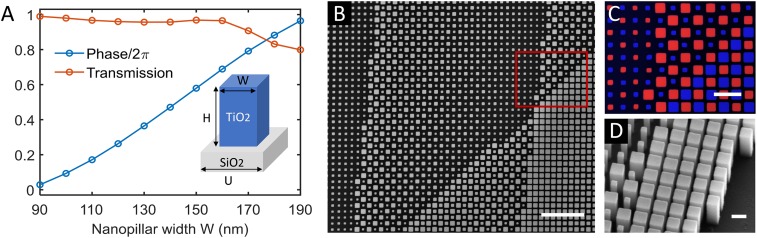
Metalens design. (*A*) Transmission efficiency and phase shift as a function of the nanopillar width. *A*, *Inset* shows the schematic of the metalens building block: a square titanium dioxide (${\text{TiO}}_{2}$) nanopillar on a glass substrate. Pillar height: H = 600 nm. Lattice unit cell size (center-to-center distance between neighboring nanopillars): U = 230 nm. By varying the pillar width (W) from 90 to 190 nm, the phase shift changes from 0 to 2π, and the transmission remains high. (*B*) Top-view SEM image of the right portion of a fabricated metalens. (Scale bar: 2 μm.) (*C*) Enlarged view of the highlighted region in *B*, with nanopillars corresponding to the 2 lens-phase profiles marked with red and blue. (Scale bar: 500 nm.) (*D*) Side-view SEM image of the edge of the metalens showing that the nanopillars have vertical sidewalls. (Scale bar: 200 nm.)

The required phase profile can be wrapped to [0,2π] (i.e., modulo 2π) without changing its functionality. Therefore, the key requirement for precise wavefront shaping is to control locally the phase between 0 and 2π. Here, we use titanium dioxide (${\text{TiO}}_{2}$) nanopillars as the building blocks for local phase control. Physically, the nanopillars function as truncated waveguides and impart a phase shift to the transmitted light. By varying the pillar width, one can tune their effective refractive index, and thus the phase shift. Fig. 3*A* shows that by changing the pillar width (W) from 90 to 190 nm, one can achieve 0 to 2π phase coverage, while maintaining a high transmission efficiency. The nanopillars have a uniform height (H) of 600 nm and can be fabricated with a single-step lithography. The center-to-center distance (U) between the neighboring nanopillars is 230 nm, smaller than half the operating wavelength. This allows us to spatially interleave different phase profiles at a subwavelength scale, which is essential to eliminating unwanted higher-order diffractions.

The metalens is fabricated with a technique demonstrated by Devlin et al. (34). Fig. 3 *B*–*D* show the scanning electron microscope (SEM) images of a fabricated sample. The location of the imaged area on the metalens is in *SI Appendix*, Fig. S6. The phase wrapping introduces a discontinuity at locations where the phase profile equals an integer number of 2π—i.e., the “zone” boundaries. This corresponds to an abrupt change of nanopillar arrangement, as shown in Fig. 3 *B*–*D*. The phase profiles of 2 off-axis lenses have different zone spacing and orientation, corresponding to the 2 nearly vertical boundaries and the diagonal boundary, respectively (Fig. 3*B* and *SI Appendix*, Fig. S6). The spatial multiplexing scheme is illustrated explicitly in Fig. 3*C*, with nanopillars belonging to different focusing profiles highlighted in different colors.

## Results

**Fig. 4.**
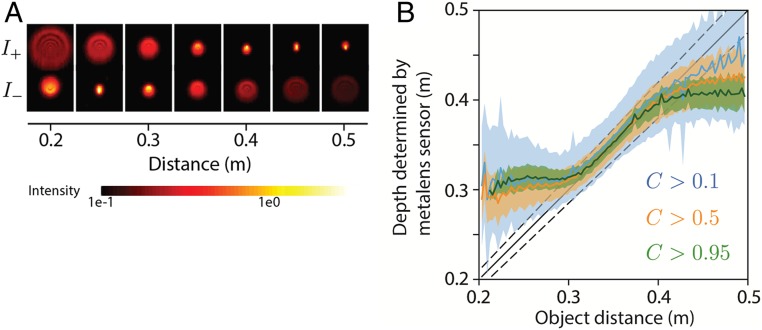
Performance analysis. (*A*) PSFs corresponding to the 2 images ($I_{+}$, $I_{-}$), measured by using green LED point light sources placed at different distances Z in front of the metalens. A spectral filter was used to limit the light bandwidth (10-nm bandwidth centered at 532 nm). The asymmetry in the PSFs results from chromatic aberration and can be eliminated by using a monochromatic laser source (*SI Appendix*, Fig. S8). (*B*) Depth Z measured by the metalens sensor as a function of known object distance. Different colors correspond to different confidence thresholds. The solid curves are the mean $\bar{Z}$ of the measured depth over many different object points that are located at the same known distance. The upper and lower boundaries of the shaded regions are the corresponding mean deviations of measured depth $\bar{\left|Z-\bar{Z}\right|}$. In obtaining both $\bar{Z}$ and $\bar{\left|Z-\bar{Z}\right|}$, only pixels whose confidence values are above the threshold are counted. The mean deviation is thus smaller for larger confidence threshold. The solid black line represents the ideal depth measurements (i.e., those equal to the known distances), and the dashed black lines represent ±5% relative differences between the measured depth and the known object distance. Within the distance range of 0.3 to 0.4 m, the measured depth is close to the ideal measurements. The mean deviation over this range is around 5% of the object distances, for a confidence threshold of 0.5. Beyond this range, the measured depth trends toward constant values that do not depend on object distance, as indicated by plateaus on the left and right. At these distances, the captured images $I_{+}$, $I_{-}$ are too blurry to provide useful contrast information for the depth measurement.

We built a prototype metalens depth sensor by coupling the metalens with off-the-shelf components. The sensor’s current size, including mechanical components such as optical mounts, is 4 × 4 × 10 cm, but since the metalens is only 3 mm in diameter, the overall size of the assembled sensor could be reduced substantially with a purpose-built photosensor and housing. We paired a 10-nm bandpass filter with the metalens, which is designed for monochromatic operation at 532 nm. A rectangular aperture was placed in front of the metalens to limit the field of view and prevent the 2 images from overlapping. The blur change between the 2 images can be seen in Fig. 4*A*, which shows PSFs for each of the 2 images [$I_{+}\left(x,y\right)$, $I_{-}\left(x,y\right)$] that were measured by using a green light-emitting diode (LED) mated to a 10-μm-diameter pinhole and placed at different depths *Z* along the optical axis. The PSFs are more disc-like than Gaussian, and they are asymmetric due to off-axis chromatic aberration. (*SI Appendix*, Fig. S8 shows that this asymmetry disappears under monochromatic laser illumination.)

To suppress the effects of noise and imaging artifacts in images ($I_{+}$, $I_{-}$), and to increase the number of high-confidence pixels in the output maps, we computed 9 separate depth and confidence maps using 9 instances of Eqs. **5** and **6** that have distinct and complementary spatial filters $F_{i}$, and then we fused these 9 “channels” into 1. We also designed a calibration procedure that tuned the parameters simultaneously, using back-propagation and gradient descent (*SI Appendix*, section 3). In addition to being user-friendly, this end-to-end calibration has the effect of adapting the computation to the shapes of the metalens PSFs, which differ substantially from Gaussians.

To analyze the depth accuracy, we measured the depths of test objects at a series of known distances and compared them with the true object distances. The test objects were textured planes oriented parallel to the lens plane. At each object distance, the mean deviation of depth, ${\text{mean}}_{x,y}|Z\left(x,y\right)-{\text{mean}}_{x,y}Z\left(x,y\right)|$, was computed by using pixels (x,y) that have confidence values greater than a threshold. Fig. 4*B* shows the measured depth for different confidence thresholds as a function of object distance. For a confidence threshold of 0.5, the measured depth was accurate to within a mean deviation below or around 5% of the true depth, over a range of true object distances between 0.3 and 0.4 m. Beyond this range, the measured depth defaulted to the extreme depth value that the system can predict, as indicated by plateaus on the left and right ends. This indicated that the 2 images were so blurry that there was an insufficient contrast difference between them.

**Fig. 5.**
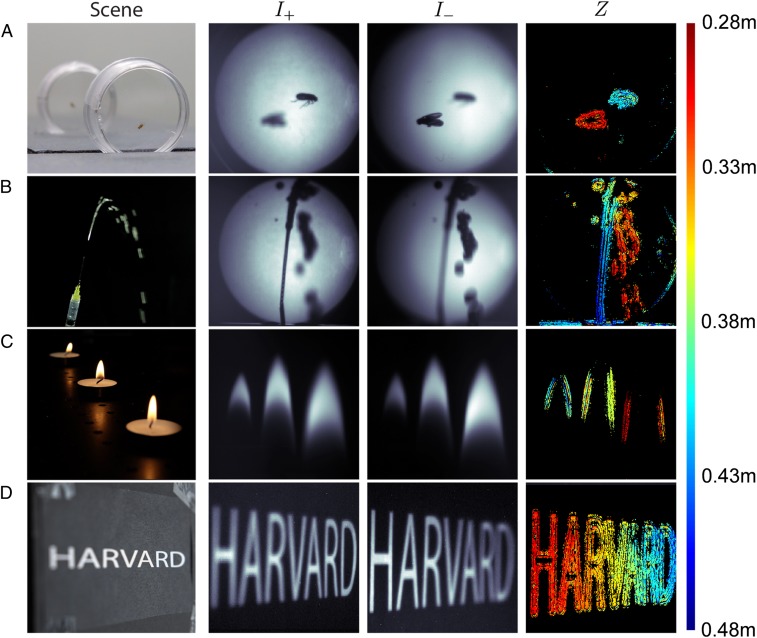
Input images and output depth maps. The sensor produces real-time depth and confidence maps of 400 × 400 pixels at >100 frames per second. (*A* and *B*) It can measure fast-moving objects such as fruit flies (*A*) and water streams (*B*) because the 2 images ($I_{+}$, $I_{-}$) are captured in a single shot instead of sequentially over time. (*B* and *C*) It can also measure translucent structures such as water streams (*B*) and flames (*C*) because it relies only on ambient light instead of reflections from a controlled light source. (*D*) A slanted plane with text expresses the difference in defocus between the 2 images ($I_{+}$, $I_{-}$). Color bar is in meters. The images and depth map for scenes *A*, *B*, and *D* were produced by illuminating the scenes with a green LED. Depth maps were thresholded at confidence greater than 0.5, which is the threshold that yields a mean deviation of about 5% of object distance between 0.3 and 0.4 m in Fig. 4*B*. Additional images and videos are available in *SI Appendix*.

Fig. 5 shows depth maps for a variety of scenes. Because it uses a single shot, the metalens depth sensor can measure objects that move, such as the fruit flies and water stream in Fig. 5 *A* and *B*. It can also measure the depth of translucent entities, such as the candle flames of Fig. 5*C*, that cannot typically be measured by using active sensors like Lidar and time-of-flight. Fig. 5*D* shows a slanted plane with printed text, where the blur change between the 2 images is particularly apparent. In general, the sensor reports a larger number of depth measurements near regions with edges and texture, whereas regions with uniform intensity and low contrast are typically discarded as having low confidence values. Note that the blur differences between $I_{+}$ and $I_{-}$ are visually apparent in Fig. 5 *A*, *B*, and *D*, but that the system still succeeds in Fig. 5*C*, where the differences in blur are hard to discern.

For scenes other than Fig. 5*C*, we used green LED light sources, and the overall transmission efficiency of the metalens plus the bandpass filter was around 15%. For sunlight illumination, the bandpass filter transmitted around 4% of the visible light of the solar spectrum. The absolute irradiance that supports the function of the sensor varied based on the sensitivity of the photosensor that was used and can be estimated from specifications including absolute sensitivity threshold, dynamic range, etc. For our experimental setup, the irradiance at the aperture was estimated to be between 0.3 and 0.5 W/m^2^ within the working bandwidth to support the function of the sensor.

The sensor generates depth and confidence maps of 400×400 pixels at more than 100 frames per second using a combined central processing unit and graphics processing unit (Intel i5 8500k and NVIDIA TITAN V). It could be accelerated substantially by optimizing the code and/or the hardware because the calculations are spatially localized and few in number. Producing the depth and confidence values at each output pixel required 637 FLOPs and involved only the 25 × 25 surrounding pixels. For context, an efficient implementation of a binocular stereo algorithm requires about 7,000 FLOPs per output pixel (37), and a system-on-chip implementation of the well-known Lucas–Kanade optical flow algorithm (with spatial dependence similar to that of our sensor) requires over 2,500 FLOPs per pixel (38).

## Discussion

The metalens depth sensor inherits some of the limitations that exist in the vision system of jumping spiders, such as a limited spectral bandwidth and a limited field of view. However, these limits are not fundamental, and they can be alleviated by more sophisticated metalens designs. The spectral bandwidth can be expanded by using achromatic metalenses (35, 39, 40), which also improve light efficiency. The field of view can be improved, for example, by using metalens nanopillars that are sensitive to polarization to induce 2 differently focused images that are superimposed on the sensor plane with orthogonal polarizations (36) and then transducing the 2 images with a spatially multiplexed, polarization-sensitive sensor array. This would effectively trade spatial resolution and light efficiency for an increase in field of view.

The proposed computational algorithm produces a dense field of depth estimates that are each associated with a confidence value. The confidence is essential for the users of the depth sensor to remove unreliable predictions. It also uses a multiscale filtering approach to handle image textures at different spatial frequency and takes advantage of the confidence to merge all different spatial scales together, compared to previous methods (20, 22) that only use filters at a single, predetermined spatial scale. The proposed algorithm does not incorporate inference-based methods such as Markov random fields (MRFs) or conditional random fields (CRFs) that could exploit longer-range coherence between depth values across the field of view. Instead, the depth and confidence estimations at each pixel are only based on information of its spatial neighborhood. The advantages of this design choice are flexibility and generality. For tasks that require high speeds, the output can be used as-is, with simple thresholding of confidence values. For tasks that require higher accuracy and fewer holes in the depth map, the current output can be fed into an MRF/CRF (or any other spatial regularizer) that is appropriate for that task. Moreover, because the pipeline is end-to-end differentiable, its parameters can be fine-tuned in conjunction with MRF/CRF parameters to optimize performance on the specific task.

By combining cutting-edge nanotechnology and computer vision algorithms, this work introduces a passive snapshot depth sensor that mimics some of the capabilities of a jumping spider. The sensor’s small volume, weight, and computation (i.e., power) bring depth-sensing capabilities closer to being feasible on insect-scale platforms, such as microrobots, ingestible devices, far-flung sensor networks, and small wearable devices. Combinations of nanophotonics and efficient computation that are different from the ones in this paper might lead to other forms of compact visual sensors, and this is an area that remains relatively unexplored (41).

## Supplementary Material

Supplementary File

Supplementary File
