# Supplementary material for: Compact single-shot metalens depth sensors inspired by eyes of jumping spiders
Source: Proc Natl Acad Sci U S A. 2019 Oct 28;116(46):22959–65. doi: 10.1073/pnas.1912154116 (PMC6859311; doi:10.1073/pnas.1912154116)
Supplement: Supplementary File [file pnas.1912154116.sapp.pdf]

PNAS
www.pnas.org

Supplementary Information for

Compact Single-Shot Metalens Depth Sensor Inspired by Eyes of Jumping Spiders

Qi Guo, Zhujun Shi, Yao-Wei Huang, Emma Alexander, Cheng-Wei Qiu, Federico Capasso, Todd Zickler

Corresponding Authors: Zhujun Shi. Federico Capasso.
Email: zhujunshi@g.harvard.edu, capasso@seas.harvard.edu

This PDF file includes:

- Statistical analysis summary
- Supplementary text
- Figs. S1 to S17
- Captions for movie S1
- References for SI reference citations

Other supplementary materials for this manuscript include the following:

- Movies S1

www.pnas.org/cgi/doi/10.1073/pnas.1912154116

Statistical analysis summary

In this paper, we report mean deviation of measured depths from true depths at pixels above certain confidence thresholds to analyze the accuracy of the depth estimation of our system. Detailed data analysis is provided in the Supplementary Information (SI) text, and the key points are summarized below:

- The definition of mean deviation, standard deviation and confidence score is provided in Sec. S1.2 and Sec. S2.2.
- Detailed training and calibration procedures are described in Sec. S3.
- Analysis of simulated data can be found in Sec. S3.2 and Fig. S13.
- Analysis of experimentally measured data can be found in Sec. S3.3, Fig. 4B and Fig. S16.

Supplementary Information Text

We propose a depth sensing platform that uses a metalens to form two adjacent images (I_+, I_-) with different amounts of defocus, as shown in Figure S1, and that efficiently computes a *depth map* comprising a measurement of the object depth at every pixel, and a *confidence map* that conveys the expected level of accuracy in the depth measurement at each pixel.

In theory, when the point spread functions of the two images are scaled Gaussian functions, and when the change in defocus between the images is small enough to be approximately differential, the depth Z is given by the expression:

$$Z = \left(\alpha + \beta \frac{F * \delta I}{F * \nabla^2 I}\right)^{-1}, \tag{S1}$$

with $\delta I = I_+ - I_-$ and $\nabla^2 I = \frac{1}{2}\nabla^2(I_+ + I_-)$, respectively, the image contrast difference and the averaged spatial Laplacians at each pixel. The linear filter F attenuates noise and optical artifacts. The scalar parameters (α, β) are determined by the dimensions of the optics (see Fig. S1):

$$\alpha = \frac{Z_{f-} + Z_{f+}}{2Z_{f+}Z_{f-}}, \tag{S2}$$

$$\beta = -\frac{1}{(\Sigma Z_s)^2}\left(\frac{1}{Z_{f+}} - \frac{1}{Z_{f-}}\right)^{-1}, \tag{S3}$$

where $Z_{f\pm}$ are in-focus distances, Z_s is the sensor distance, Σ is the entrance pupil size. These equations are equivalent to equation (5) in the main paper.

We also design a confidence score to indicate the reliability of the depth measurement at each pixel:

$$C = f(|\gamma_1 \delta I + \gamma_2 (\nabla^2 I)^{-1} + \gamma_3|) \in (0,1), \tag{S4}$$

where $(\gamma_1, \gamma_2, \gamma_3)$ are confidence parameters that depend on the dimensions of the optics, and $f(\square)$ is a nonlinear function that normalizes the confidence value to the range $[0,1]$ (to be described later in Section 0). Low confidence occurs, for example, in regions where the images (I_+, I_-) have uniform intensity and low contrast, subjecting the contrast difference δI and second order derivative $\nabla^2 I$ to excessive noise.

This supplementary material provides details about the system. Section 0 describes the calculations for depth and confidence, and it analyzes the effects of the rectangular aperture used to prevent overlap between the two side-by-side images. Section 2 provides details about the hardware and algorithm. Section 0 describes the calibration process and elaborates on evaluation.

1. Analysis

1. Depth from differential defocus. For completeness, we include the following derivation of the depth equation. It is adapted from Guo et al. (1), who introduced the equation it in the context of a traditional refractive lens that deforms over time. Consider a thin lens camera with sensor distance Z_s, in-focus distance Z_f, and entrance pupil size Σ

taking a picture of a front parallel plane placed at distance Z from the lens. The captured image $I(x, y)$ at sensor plane location (x, y) is:

$$I(x, y; Z, Z_s, Z_f, \Sigma) = h(x, y; Z, Z_s, Z_f, \Sigma) * T(x, y), \tag{S5}$$

where $h(x, y, Z)$ is the point spread function (PSF) that we model as a Gaussian function

$$h(x, y; Z, Z_s, Z_f, \Sigma) = \frac{1}{2\pi\sigma^2(Z, Z_s, Z_f, \Sigma)} \exp\left(-\frac{x^2 + y^2}{2\sigma^2(Z, Z_s, Z_f, \Sigma)}\right) \tag{S6}$$

with standard deviation σ, and T is the sharp image of the scene as if the camera is a pinhole. See Fig. S2. According to the thin-lens equation

$$\frac{1}{f} = \frac{1}{Z_f} + \frac{1}{Z_s} = \frac{1}{Z} + \frac{1}{Z'}, \tag{S7}$$

where f is the focal length of the lens, and Z' is the distance from the entrance pupil to the focused light from the object (Fig. S2). By similar triangles, the PSF standard deviation σ and the entrance pupil size Σ are related by:

$$\frac{\sigma}{Z_s - Z'} = \frac{\Sigma}{Z'}. \tag{S8}$$

Combining equation (S7) and (S8) yields the equation of PSF standard deviation σ:

$$\sigma(Z, Z_s, Z_f, \Sigma) = \left[\left(\frac{1}{Z_f} - \frac{1}{Z}\right) Z_s\right] \Sigma. \tag{S9}$$

Taking derivatives of both sides of equation (S5) with respect to the in-focus distances Z_f while keeping other optical parameters fixed yields:

$$\frac{\partial I(x, y; Z, Z_s, Z_f, \Sigma)}{\partial Z_f} = \frac{\partial h(x, y; Z, Z_s, Z_f, \Sigma)}{\partial Z_f} * T(x, y),$$

where $\partial h / \partial Z_f$ is shown in the main paper (equation (3)) to have the following property:

$$\frac{\partial h(x, y; Z, Z_s, Z_f, \Sigma)}{\partial Z_f} = \frac{\partial \sigma(Z, Z_s, Z_f, \Sigma)}{\partial Z_f} \cdot \sigma(Z, Z_s, Z_f, \Sigma) \cdot [\nabla^2 h(x, y; Z, Z_s, Z_f, \Sigma)].$$

The two above equations jointly give

$$\frac{\partial I(Z_f)}{\partial Z_f} = \frac{\partial \sigma(Z_f)}{\partial Z_f} \cdot \sigma(Z_f) \cdot \nabla^2 I(Z_f). \tag{S10}$$

For simplicity, from this point forward we omit notation for the sensor location (x, y) and for optical parameters that are constants. For any sort of camera that provides control of in-focus distance Z_f while keeping all other optical dimensions fixed, we can measure $\frac{\partial I(Z_f)}{\partial Z_f}$, $\frac{\partial \sigma(Z_f)}{\partial Z_f}$, and $\nabla^2 I(Z_f)$ in equation (S10) via finite differences:

$$\frac{\partial I(Z_f)}{\partial Z_f} = \frac{I(Z_f + \delta Z_f) - I(Z_f - \delta Z_f)}{2\delta Z_f},$$

$$\frac{\partial\sigma(Z_f)}{\partial Z_f}=\frac{\sigma(Z_f+\delta Z_f)-\sigma(Z_f-\delta Z_f)}{2\delta Z_f},$$

$$\nabla^2 I(Z_f)=\nabla^2\left[\frac{I(Z_f+\delta Z_f)+I(Z_f-\delta Z_f)}{2}\right], \tag{S11}$$

where $I(Z_f)$ indicates the image taken with in-focus distance Z_f, and $\delta Z_f \ll Z_f$ is a differential change of in-focus distance. Using these measurements we can solve for the standard deviation of the PSF σ, and thus depth Z, via equation (S10) and (S5), in closed form:

$$Z=\left(\frac{1}{Z_f}+\frac{Z_f^2}{(\Sigma Z_s)^2\delta Z_f}\cdot\frac{I(Z_f+\delta Z_f)-I(Z_f-\delta Z_f)}{\nabla^2[I(Z_f+\delta Z_f)+I(Z_f-\delta Z_f)]}\right)^{-1}. \tag{S12}$$

In this work, we build a metalens imaging system that simultaneously creates a pair of images of the same scene through a shared aperture with two different in-focus distances $Z_f \mp \delta Z_f$, and that uses the image pair to compute depth via equation (S12). The system is depicted in Fig. S1. Denoting the image pair as $I_{\pm}=I(Z_f \mp \delta Z_f)$ and the in-focus distances as $Z_{f\pm}=Z_f \mp \delta Z_f$, equation (S12) can be simplified to:

$$Z=\left(\alpha+\beta\frac{\delta I}{\nabla^2 I}\right)^{-1}, \tag{S13}$$

where $\alpha=\frac{Z_{f-}+Z_{f+}}{2Z_{f+}Z_{f-}}, \beta=-\frac{1}{(\Sigma Z_s)^2}\left(\frac{1}{Z_{f+}}-\frac{1}{Z_{f-}}\right)^{-1}$, $\delta I=I_+-I_-$, and $\nabla^2 I=\frac{1}{2}\nabla^2(I_+ + I_-)$. Parameters α,β are constants determined by the optics, whereas δI and $\nabla^2 I$ can be measured from images (I_+, I_-).

There is an analogy between this depth from differential defocus equation and the classical diffusion process. Rewriting equation (4) in the main text as

$$\frac{\partial I}{\partial\sigma^2}=\frac{1}{2}\nabla^2 I, \tag{S14}$$

one recognizes that it is identical to the two-dimensional diffusion equation, $\partial u/\partial t = D\nabla^2 u$, where the PSF variance σ^2 plays the role of diffusion time t; the diffusion constant D equals ½; and the image intensity I corresponds to the particle concentration u. The impulse response of the 2D diffusion equation is $u(t)=\frac{1}{4\pi Dt}\exp\left(-\frac{x^2+y^2}{4Dt}\right)$, which is the same as the Gaussian PSF in equation (S6). Physically, the analogy reflects the fact that the image blur is a local averaging process, where local energy conservation and linear restoring flux define the system dynamics.

Although Gaussian PSFs are required to derive equation (S10) in theory(2), we experimentally find that images generated using non-Gaussian PSFs can still be used to predict depth through equation (S13) following the calibration process described in Section 0. Experimentally, we also find that the system can handle surfaces that deviate from being front parallel, as long as the PSFs are locally constant across the spatial supports ("receptive fields") of the output pixels.

2. Depth error and confidence. If we assume i.i.d. additive Gaussian noise in the captured images with zero-mean and variance ϵ^2, the image contrast difference δI and second order derivative $\nabla^2 I$ are also independent and Gaussian-distributed:

$$\delta I \sim N(\delta I^*, \epsilon^2), \quad \text{(S15)}$$

$$\nabla^2 I \sim N(\nabla^2 I^*, \|\nabla^2\|^2 \epsilon^2), \quad \text{(S16)}$$

with mean values δI^* and $\nabla^2 I^*$ that depend on the object being imaged. In practice we approximate the derivatives with finite differences, thus the symbol ∇^2 here represents the second order differential filter, and the operation $\|\nabla^2\|$ computes the two-norm of it. We can use this model to derive an estimate of the standard deviation of Z that is computed by equation (S1) under the noise model of equations (S15) and (S16), and we can use the inverse of this quantity as our confidence score. A first-order Taylor expansion of equation (S1) gives the standard deviation(1):

$$StdZ \approx \left(\frac{E_1^2}{E_2^2}\left(\frac{V_1}{E_1^2} + \frac{V_2}{E_2^2} - \frac{2V_3}{E_1 E_2}\right)\right)^{\frac{1}{2}}, \quad \text{(S17)}$$

where $E_1 = F * \nabla^2 I^*$, $E_2 = \alpha F * \nabla^2 I^* + \beta F * \delta I^*$, $V_1 = \|F * \nabla^2\|^2 \epsilon^2$, $V_2 = (\alpha^2 \|F * \nabla^2\|^2 + \beta^2 \|F\|^2)\epsilon^2$, $V_3 = \alpha \|F * \nabla^2\|^2 \epsilon^2$. Since ∇^2 and F are both filters, $\|F * \nabla^2\|$ denotes the 2-norm of their convolution. We assume $F = [1]$ to be the identity filter here for simplicity.

The measured intensity noise level of the sensor in our system is approximately $\epsilon = 0.7LSB$ (least significant bit). Fig. S3a plots the standard deviation of depth Z as a function of measurements $|1/\nabla^2 I^*|$ and $|\delta I^*|$ based on equation (S17) and this measured noise level ϵ. Given a scene such as Fig. S3b, we could compute the standard deviation of Z at every pixel point by estimating the mean values $|1/\nabla^2 I^*|$ and $|\delta I^*|$ and using Fig. S3a as a look up table. In practice, we simply set the mean values $|1/\nabla^2 I^*|$ and $|\delta I^*|$ equal to the measured ones $|1/\nabla^2 I|$ and $|\delta I|$. In Fig. S3b, the colored crosses indicate the standard deviations of depth Z for three different image points indicated by the corresponding crosses in Fig. S3a. Textureless image regions (blue cross in Fig. S3b) generally have high standard deviation, while those with substantial contrast have low standard deviation.

The shape of the surface in Fig. S3a suggests that the standard deviation of depth Z can be approximated by a linear function of the two variables $(|1/\nabla^2 I|,\ |\delta I|)$. Based on this, we propose a simple linear function s_Z to fit the standard deviation of Z,

$$s_Z = |\gamma_1 |\delta I| + \gamma_2 |(\nabla^2 I)^{-1}| + \gamma_3|, \quad \text{(S18)}$$

and define our confidence score C as an inversion of this that is normalized to the range $(0,1)$:

$$C = f(s_Z) \in (0,1). \quad \text{(S19)}$$

The normalization function $f(\square)$ is non-parametric. It normalizes the standard deviation s_Z to the range $(0,1)$, where a higher confidence score C corresponds to a lower standard deviation s_Z. We choose as the normalization function $f(s_Z) = 1 - g(s_Z)$ with $g(s_Z)$ a piecewise linear fit to the normalized cumulative histogram of a large set of per-pixel

standard deviations $\{s_Z^i\}$ that result from applying equation (S18) to a pre-defined dataset of input images.

3. Aperture and Vignetting. As shown in Fig. S1, the sensor incorporates a rectangular aperture to prevent the two images of I_+ and I_- from overlapping. Other shapes, such as circular apertures, could also be used. In addition to preventing overlap, the aperture has the effect of reducing light collection efficiency for off-axis incident angles. We characterize this vignetting effect here.

Depending on the location and the size of the aperture, there are two possible scenarios: (1) the metalens is the entrance pupil of the system (Fig. S4a), and there is no additional loss of light collection efficiency due to the aperture for on-axis objects; (2) the rectangular aperture is the entrance pupil of the system (Fig. S4b), and the on-axis light collection efficiency is limited by the aperture. It is clear that for maximum efficiency, scenario (1) is more desirable. This is also the case in our system.

Although in scenario (1) there is no additional on-axis loss, the aperture may block some of the light for off-axis objects (Fig. S5ab). To quantify this effect, we calculated the light collection efficiency for various field angles.

In our system, the distance Z between the object and the metalens is much larger than the metalens radius r ($Z \approx 150\,mm \gg r = 3mm$). Therefore, we can use small-angle approximation and assume that the light intensity is approximately uniform across the metalens without the aperture. The introduction of the aperture blocks some of the light, leaving a shadow on the metalens. The shape of the aperture is rectangular, so the shadow has a linear boundary (Fig. S5c). The size of the bright area (A_{bright}) determines the collection efficiency. Note that due to the rectangular shape of the aperture, the bright area is a circular segment. Its height (sagitta) is given by

$$\xi = Z\Delta = Z\{\min(\zeta_1, \zeta_2) - \max(\eta_1, \eta_2)\}, \qquad (S20)$$

where here again we use the small-angle approximation and drop the factor of $\cos^2\theta$, θ being the field angle. Δ is the tangential angular range of light that can be captured by the system. The angles ζ_1, ζ_2 and η_1, η_2 are as depicted in Fig. S5b. It can be shown that,

$$\eta_1 = \text{atan}\left(\frac{Z\tan\theta - r}{Z}\right); \quad \eta_2 = \text{atan}\left(\frac{Z\tan\theta - l\tan\psi}{Z-l}\right); \quad \zeta_1 = \text{atan}\left(\frac{Z\tan\theta + r}{Z}\right); \quad \zeta_2 = \text{atan}\left(\frac{Z\tan\theta + l\tan\psi}{Z-l}\right), \quad (S21)$$

where l is the distance between the rectangular aperture and the metalens, and ψ is half the angular size of the aperture relative to the metalens center.

Finally, the bright area and collection efficiency are, respectively, given by:

$$A_{\text{bright}} = r^2 \,\text{acos}\frac{r-\xi}{r} - (r-\xi)\sqrt{r^2 - (r-\xi)^2} \qquad (S22)$$

$$\chi \equiv \frac{\text{Light collection efficiency with aperture}}{\text{Light collection efficiency without aperture}} = \frac{A_{\text{bright}}}{\pi r^2}, \qquad (S23)$$

If the collection efficiency χ is equal to 1, no areas on the metalens is occluded by the aperture. Fig. S5d shows the relative light collection efficiency χ as a function of incident

angle. Each line corresponds to an aperture located at a different distance but with the same angular size $\psi = 2.3°$, which is similar to the dimensions of our prototype. An aperture placed further from the metalens induces less vignetting, and conversely, when the aperture is placed closer to the metalens, the vignetting effect is more significant. This will add a low frequency, asymmetrical variation of intensity on the captured image pair (I_+, I_-), that can be effectively removed by our algorithm discussed in Section 0.

2. Implementation details

1. Optics. Our prototype consists of a custom-built rectangular aperture that limits the field of view to avoid overlap between the images (I_+, I_-). It also pairs the 3mm-diameter, custom-built metalens with a bandpass filter (FL532-10, Thorlabs, Inc.) that limits the full-width-at-half-maximum (FWHM) of the incoming light spectrum to 10nm centered at 532nm, and a monochrome photosensor (Grasshopper 3 GS3-U3-23S6M-C, FLIR) with a global shutter and a maximum frame rate of 160 frames-per-second. The dimensions of the system are about 4cm×4cm×10cm, but since the diameter of the lens is only 3mm and the thickness of the metalens (together with the glass substrate) is only 1.5 mm, its size could be substantially reduced using special-purpose components.

As discussed in Section 0, the aperture is not necessarily rectangular, and can be any shape as long as the image pair does not overlap. The vignetting effect introduced by the aperture can be effectively removed using appropriate image filters.

As shown in Fig. S1, the metalens uses spatial multiplexing to incorporate two off-axis lens phase profiles within a shared aperture. Before they are multiplexed, each off-axis lens phase profile is designed to modulate an incident spherical wavefront, which is at wavelength λ and is centered along the optical axis at the in-focus plane, so that all light arrive at the off-axis point of focus on the sensor in phase. Each of the two phase profiles, $\phi_+(x,y)$ and $\phi_-(x,y)$, has a distinct in-focus distance, Z_{f+} and Z_{f-}, respectively, and the points of focus are at symmetric transverse offsets $\pm D$ from the optical axis. The phase profiles that satisfy these criteria are:

$$\phi_\pm(x,y) = -\frac{2\pi}{\lambda}\left(\sqrt{x^2+y^2+Z_{f\pm}^2} + \sqrt{x^2+(y\mp D)^2+Z_s^2} - \sqrt{D^2+Z_s^2} - Z_{f\pm}\right), \text{(S24)}$$

where as shown in Fig. S1, $Z_{f\pm}$ is the designed in-focus distance, Z_s is the distance between the metalens and photosensor, and D is the transverse displacements of the off-axis image centers. The shapes of $\phi_\pm$ are shown in Fig. S6a-b. In our design, the dimensions are $Z_{f-} = 18\ cm$, $Z_{f+} = 14.4\ cm$, $Z_s = 4\ cm$, and $D = 1.5\ mm$, and the working wavelength is $\lambda = 532nm$. The overall phase profile is achieved by interleaving the two phase profiles at a subwavelength scale.

The assembled sensor distance Z_s' can be different from the designed one Z_s, which results in different assembled in-focus distances $Z_{f\pm}'$. All that is required is an adjustment to the two parameters (α, β) that appear in the equation for depth (equation (S1)), so that the depth equation changes to:

$$Z = \left(\nu_1 + \nu_2\left(\alpha + \beta\frac{F * \delta I}{F * \nabla^2 I}\right)\right)^{-1}, \qquad (S25)$$

with two suitable "adjustment" parameters (ν_1, ν_2). The optical parameters (α, β) are changed to $(\nu_1 + \nu_2\alpha, \nu\beta)$ to accommodate the difference in dimensions between the designed and the assembled system.

We perform the following calibration steps to crop and align the image pairs (I_+, I_-) that are transduced at the photosensor. We place a point light source in front of the camera, which generates two bright spots on (I_+, I_-) that are identified by the locations of the centers. By repeating this as we vary the location of the point light source, we obtain a dense set of corresponding 2D points between I_+ and I_-. Using these correspondences, we can find out a linear perspective transformation (homography), which is represented by a 3×3 invertible matrix H, that maps each 2D pixel location in I_+ to its corresponding point in I_- and thereby aligns the two images. We experimentally find that matrix H does not change with the depth of the point source, and that overall, the alignment error is less than one pixel. A typical measurement from the photosensor, along with a superimposed visualization of the points used for alignment, are shown in Fig. S7.

2. Computational architecture. The filter F in the depth equation (equation (S1)) can be used to improve the quality of the depth measurement by attenuating sensor noise. It is also beneficial to compute separate depth measurements using distinct filters and then merge these measurements into a final depth map(1). This is evident from the noise analysis of Section 0, which implies that the variances of two depth measurements at a single pixel obtained using two different filters F_i and F_j will be determined by the values of the filtered contrast differences and second order derivatives, $(F_i * \delta I, F_i * \nabla^2 I)$ and $\left(F_j * \delta I, F_j * \nabla^2 I\right)$, which, if the filters are properly designed, can be complementary: When one depth measurement has high variance (low confidence), the other can have low variance (high confidence).

For the metalens depth sensor, we use a set of nine filters $\{F_i\}$ $(i = 1 \dots 9)$ having different shapes and spatial supports, which provides a balance between depth accuracy and computational complexity. The contrast difference and the second order derivative from each filter $(F_i * \nabla^2 I, F_i * \delta I)$ are used to individually generate an estimate. These estimates are fused together by probabilistic inference. This computation is end-to-end differentiable, therefore the parameters in the network can be automatically tuned instead of manual calibration, using back-propagation and stochastic gradient descent, to optimize the depth accuracy for a simulated set of natural-looking objects. The remainder of this section describes the sequence of calculations of our algorithm, arranged in a feed-forward computational graph, that compute depth and confidence using multiple filters; and the next section describes how the parameters are automatically tuned to optimize depth accuracy.

Fig. S9a shows the full computational graph of the metalens depth sensor. In addition to depth map Z, it also produces a confidence map C that indicates the expected accuracy of the depth at each pixel. For simplicity, the computational graph predicts the inverse depth $P = 1/Z$, in the single-filter case using:

$$P = \alpha + \beta \frac{F * \delta I}{F * \nabla^2 I}, \tag{S26}$$

and then calculates the inverse of this to obtain depth Z.

The first operations applied to the (aligned) input images (I_+, I_-), are the spatial second order derivative and the pixel-wise contrast difference:

$$\nabla^2 I = \frac{1}{2}\nabla^2 * (I_+ + I_-), \tag{S27}$$

$$\delta I = (I_+ - I_-). \tag{S28}$$

We use this filter to estimate the Laplacian:

$$\nabla^2 = \begin{bmatrix} 0 & 0.0013 & 0.004 & 0.0013 & 0 \\ 0.0013 & 0.0377 & 0.1162 & 0.0377 & 0.0013 \\ 0.004 & 0.1162 & -0.6421 & 0.1162 & 0.004 \\ 0.0013 & 0.0377 & 0.1162 & 0.0377 & 0.0013 \\ 0 & 0.0013 & 0.004 & 0.0013 & 0 \end{bmatrix}. \tag{S29}$$

A bank of nine pre-determined filters F_i are separately convolved with the second order derivatives $\nabla^2 I$ and the contrast difference maps δI to produce nine tuples of $\{(F_i * \nabla^2 I, F_i * \delta I)\}$. Each tuple generates an estimate of inverse depth P^i using a robust version of equation (S26):

$$P^i = \alpha^i + \beta^i \frac{(F_i * \delta I)(F_i * \nabla^2 I)}{(F_i * \nabla^2 I)^2 + \rho_1}, \tag{S30}$$

with the stabilizing constant set to $\rho_1 = 10^{-5}$.

For the confidence map, we first calculate an approximation of the standard deviation of each inverse depth P^i. The first order approximation of equation (S1) along with equation (S26) yields:

$$Z \approx \frac{1}{\alpha}\left(1 - \frac{\beta \delta I}{\alpha \nabla^2 I}\right) = \frac{2}{\alpha} - \frac{P}{\alpha^2} \propto P, \tag{S31}$$

which indicates the standard deviation of inverse depth P is proportional to the standard deviation of Z. Thus, the approximate standard deviation of P uses a form similar to equation (S18):

$$s_{P^i} = \left| \gamma_3^i + \sum_{j=1}^{9} \gamma_1^{ij} |\delta I^j| + \gamma_2^{ij} \frac{1}{|\nabla^2 I^j| + \rho_2} \right|, \tag{S32}$$

where $\{(\gamma_1^i, \gamma_2^{ij}, \gamma_3^{ij})\}$ are tunable coefficients and ρ_2 is another stabilizing constant, $0 < \rho_2 \ll |\nabla^2 I|$ that is set to $\rho_2 = 10^{-1}$ in our implementation.

Finally, each estimate of inverse depth is interpreted as a Gaussian distribution, with mean P^i and standard deviation s_{P^i}, and the nine estimates are fused into a maximum likelihood estimate:

$$P = mean_j\, P^j, \tag{S33}$$

$$s_P = \left(mean_{\mathrm{j}}(s_{P^j})^2 + mean_{\mathrm{j}}(P^j)^2 - P^2\right)^{\frac{1}{2}}. \tag{S34}$$

Two tunable parameters ν_1 and ν_2 are included to allow adjustment of the effective sensor-lens distance Z_s as described in equation (S25), and thus the final depth is given by:

$$Z = (\nu_1 + \nu_2 P)^{-1}. \tag{S35}$$

As described in Section 0, the coefficients ν_1 and ν_2 can be adjusted whenever the system's sensor distance is modified. The final confidence score is:

$$C = f(s_P). \tag{S36}$$

The filter banks $\{F_i\}$ we use in the computational graph is shown in Fig. S9b. Each filter in this bank has the analytic form:

$$F(x, y; \kappa, m, n) = \frac{\partial^{m+n}}{\partial^m x \partial^n y}\left(\exp\frac{-(x^2+y^2)}{2\kappa^2}\right) * \left(1 - \exp\frac{-(x^2+y^2)}{2\kappa_{low}^2}\right), \tag{S37}$$

where κ determines the scale of the filter and m, n control the shape, and the symbol $*$ denotes the two dimensional spatial convolution. Each filter is a derivative of Gaussian multiplied by a high pass filter with standard deviation κ_{low} that has the effect of eliminating the low-frequency vignetting effect described in Section 0. Note that the multi-scale Gaussian component of the filter bank together with the image second order derivative can be efficiently implemented using the scheme proposed by Burt and Adelson(3).

The full feed-forward computational graph is shown in Fig. S8a, and it includes 191 tunable parameters:$\{\alpha^i, \beta^i\}, \{(\gamma_1^i, \gamma_2^{ij}, \gamma_3^{ij})\}, \nu_1, \nu_2$. Since the computational graph is feed-forward, and since the final depth and confidence values that it produces are differentiable with respect to each parameter, all of the parameters can be automatically optimized by back-propagation and gradient descent to optimize the depth prediction accuracy of a dataset of scenes that have known depth. This property of the computational graph makes calibration particularly convenient and avoids requiring precise optical positioning of the components.

3. Training and Calibration

Training and calibrating the computational graph means finding values for the tunable parameters that produce depth measurements that are as accurate and confident as possible. In order to minimize the workload each time a new system is assembled, we divide the process into two steps. First we tune the majority of parameters $\{\alpha^i, \beta^i\}, \{(\gamma_1^i, \gamma_2^{ij}, \gamma_3^{ij})\}$ in simulation. Then we adjust the rest two parameters (ν_1, ν_2) using images that are captured by the physical instantiation of the sensor.

1. Rendering defocused images for training. To perform simulation, we built a rendering system that simulates defocused image pairs (I_+, I_-) from a digital description of a virtual three-dimensional scene. The rendering system represents the metalens by a tabulated set of measured point spread functions (PSFs), like the ones in Fig. S8.

Fig. S10a depicts our rendering process in two dimensions for illustration purposes. In practice it operates in three dimensions using planar segments instead of linear ones. Our renderer accepts as input a collection of tabulated PSF that are either specified, or measured by placing a point light source in front of the sensor, at a discrete set of spatial locations

$\{(X_k, Z_k)\}$. We denote the PSF for any source location (X, Z) as $k(u; X, Z)$, where u indexes the pixels on the photosensor with origin at the chief ray. For simplicity, we describe here the rendering for a single slanted line segment; more complicated shapes can be decomposed into piecewise linear segments.

As is shown in Fig. S10b, our system first approximates the PSF at each vertex (endpoint) of the line segment $\{(X^v, Z^v)\}_{v=0,1}$, denoted as $k(u; X^v, Z^v)$, using bilinear interpolation between the grid locations at which the PSF was specified/measured. Then, the PSF $k(u; X, Z)$ at every point (X, Z) on the segment is approximated using linear interpolation of PSFs at each vertex (X^v, Z^v) with weight $w_v\left(\frac{X}{Z}\right)$, as in Fig. S10c. Finally, the image $I(x)$ is the summation of the (interpolated) PSFs at all points on the line segment, weighted by the spatial texture (emitted radiance) pattern that exists on the segment:

$$I(x) \approx \sum_{X,Z} \sum_{v} k\left(x - \frac{X}{Z}; X^v, Z^v\right) w_v\left(\frac{X}{Z}\right) P\left(\frac{X}{Z}\right)$$
$$= \sum_{v} k(x; X^v, Z^v) * \big(w_v(x) T(x)\big), \qquad \text{(S38)}$$

where $T(x)$ denotes the texture (emitted radiance) at the point that projects to $(x, 1)$ on the image plane. In practice, we enhance the accuracy of this approach by sampling the specified/measured PSFs more finely, i.e., by using a finer sampling of point source locations (X_k, Z_k), in regions of the space where the system is more focused and the PSFs are sharper.

As described so far, the rendering process applies to any continuous, piecewise-linear surface. During parameter tuning we also want the computational system to experience scenes that contain discontinuities in surface depth, so that it can learn to associate the nearby image points with low predicted confidence values. Depth discontinuities create occlusion events, meaning that some rays through the metalens aperture see surface points that other rays do not. These effects are hard to be modeled exactly using only PSFs of the optical system, but for piece-wise linear scenes, we can simulate a close approximation as follows.

We divide the (possibly discontinuous) scene into piecewise linear segments $l = 1, \ldots, N$, and for each segment l we separately render its image $I_l(x)$ on the photosensor using equation (S38). We also render a blur mask $M_l(x)$ that is an image of segment l with constant texture $T(x) = 1$. Finally, we sum all the images of segments together, weighted by the blur masks of the foreground segments:

$$I(x) = \sum_{l} I_l(x) \prod_{s \text{ occludes } l} \big(1 - M_s(x)\big). \qquad \text{(S39)}$$

Fig. S11 shows a sample image I corresponding to a typical scene shape Z_{true} that is generated by our rendering system. It contains slanted, planar foreground and background segments, with depth discontinuities along the foreground-background boundary. Fig. S11 also shows the segmentation of the shape, the rendered image I_l and the blur mask M_l of each segment l, as well as the final rendered image I. When the system renders the image pairs (I_+, I_-) required by the metalens sensor, two tabulated sets of PSFs are provided to

separately render the two differently defocused images for each scene shape. The two sets of provided PSFs were Gaussian functions that have standard deviations fitted to designed PSFs of the metalens depth sensor. Experimentally, we observe that our layer-based approximation to the defocusing effects adjacent to depth discontinuities performs better than alternative approximations, such as applying blur to an all-in-focus pinhole image(4).

2. Training with simulated data. Using the rendering system we generate a dataset of 500 tuples $(I_+, I_-, Z_{\text{true}})$ of randomly-generated, two-layer scenes. For these, we randomize the slants and tilts of the foreground and background segments, and we randomly generate triangular foreground boundary shapes. The textures for the foreground and background scene planes are randomly selected, from a database of calibrated photographs of textures under a variety of lighting conditions(5).

Using this dataset, we perform back-propagation and gradient descent to automatically tune the values of the computational parameters in order to optimize the 1-norm loss function:

$$L(Z, C, Z_{\text{true}}) = mean\big(err(Z, Z_{\text{true}}) \cdot W(Z_{\text{true}})\big) \tag{S40}$$

where $err(\cdot,\cdot)$ is an error based on inverse depth,

$$err(Z, Z_{\text{true}}) = \left|\frac{1}{Z} - \frac{1}{Z_{\text{true}}}\right|, \tag{S41}$$

that experimentally results in fewer outliers than using an error based on depth $|Z - Z_{true}|$, and W gives higher weights to pixels that are close to depth discontinuities. In practice, we use:

$$W(x, y) = \begin{cases} 5, & (x, y) \text{ is within 9 pixel from a depth boundary} \\ 1, & \text{otherwise} \end{cases}, \tag{S42}$$

which is visualized in Fig. S14.

We use the Adam optimizer, with learning rate 0.001, to train parameters $\{\alpha^i, \beta^i\}, \{(\gamma_1^i, \gamma_2^{ij}, \gamma_3^{ij})\}$ at the same time, while fixing the rest two parameters $(\nu_1 = 0, \nu_2 = 1)$. The training takes in an image pair (I_+, I_-) at each iteration (batch size=1). Convergence occurred after roughly 4000 iteration, and the total training time is 586 secs. The training and the validation losses across iterations are shown in Fig. S12. We use 400 out of the 500 simulated scenes for training, and the rest for validation.

Fig. S13a-c provides a quantitative analysis of the depth accuracy on the simulated validation set. Fig. S13a shows the distribution of depth measurements as a function of object distance from the sensor ("true depth"). Most measurements have less than 10% relative error (dashed line). Fig. S13b shows a *sparsification plot*, which summarizes how well the system can exploit different confidence thresholds to trade between outputting sparse depth maps that are accurate and dense depth maps that are less accurate. The abscissa is the sparsity, or fraction of output pixels at which depth is reported, and the red curve is the confidence threshold that produces each sparsity value. Meanwhile, the black curve is the mean error of the depth measurements that are reported. Fig. S13c plots the mean error as a function of object distance for the three different confidence thresholds that are indicated by the vertical dashed lines in Fig. S13b.

3. Fine tuning with captured data. Following the initial round of training with rendered data, we adjust the values of the two coefficients (ν_1, ν_2) using images (I_+, I_-) that are captured by the metalens depth sensor. For this, we fabricate flat, textured, planar objects and align them in front of the sensor such that the depth Z_{true} at every pixel is known. We use grid search to find the two parameter values that minimize a robust objective:

$$L_{\text{fine tune}}(Z, Z_{true}) = \begin{cases} err(Z, Z_{true}), & err(Z, Z_{true}) < 0.02 \\ 0.02, & \text{otherwise} \end{cases}. \quad \text{(S43)}$$

The textures are randomly drawn from Describable Textures Dataset(6) and are printed and glued onto flat planar objects (see Fig. S15 for examples). We use two for training and ten for testing. For each textured plane, we align it to be parallel to the sensor plane and move it to different depths Z_{true} between 0.1 and 0.4m with step size 0.01m.

Similar to the validation, the quantitative testing result is shown in Fig. S16a-c. We find that the system can predict depths over the interval [0.3m, 0.4m], with depth errors that are smaller than 10% of the true depth. Increasing the confidence threshold reduces error (in exchange for lower density), and the overall mean errors of the 50% most confident and the 5% most confident pixels, respectively, are about 0.04m and 0.03m respectively.

Fig. S17 shows additional examples of depth maps that are produced by the sensor for different scenes. Fig. S17a is an “infinite mirror” consisting of two mirrors that reflect light from LED light sources. In this case, the sensor recovers the depth of the LED as well as the depths of all virtual images of that LED. Fig. S17b shows one view of a finger gesture, suggesting a possible application to gesture-based human-computer interfaces on small, low-power platforms like wearables.

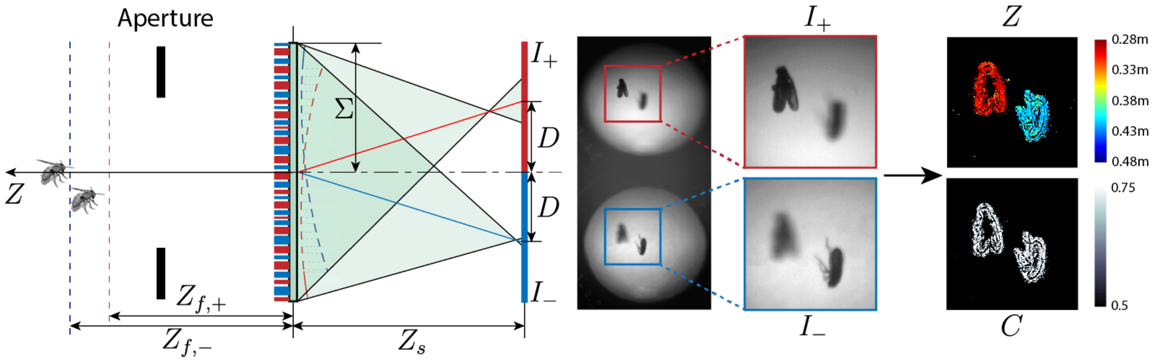

Fig. S1. System pipeline. The metalens depth sensor captures two images (I_+, I_-) through the same aperture that have different in-focus distances (Z_{f+}, Z_{f-}). A sequence of calculations applied to each local neighborhood of these images produces a per-pixel depth map Z and a per-pixel confidence map C.

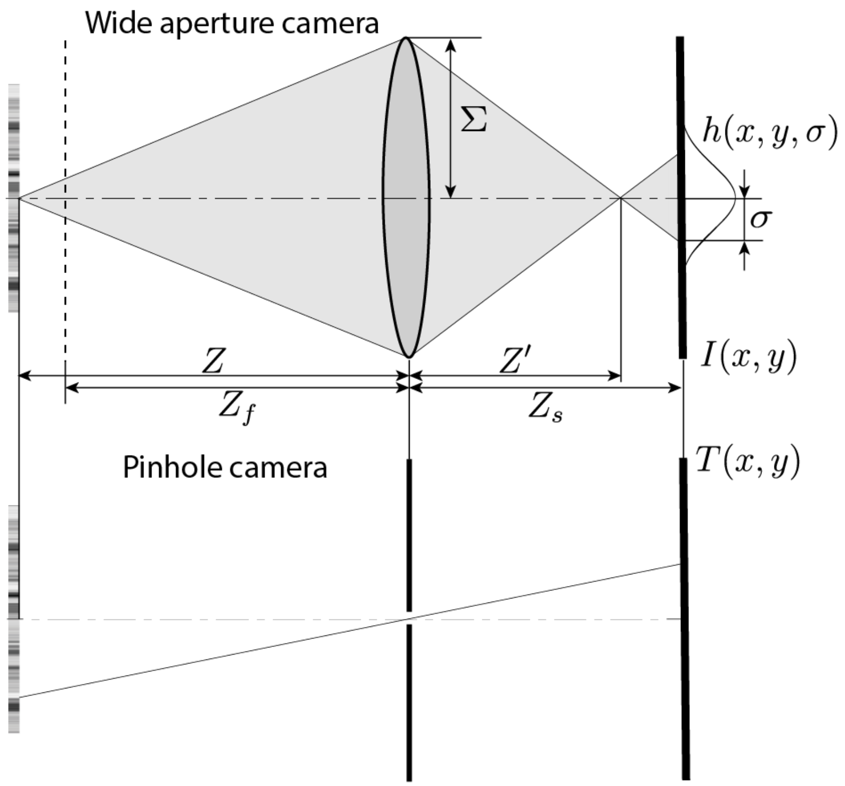

Fig. S2. Wide aperture camera and pinhole camera model. When imaging a front-parallel plane using a wide aperture camera, the image formed on the photosensor $I(x, y)$ is the convolution of the point spread function $h(x, y, \sigma)$ with the image $T(x, y)$ of the same scene as if taken by a pinhole camera with the same sensor distance (equation (S5)).

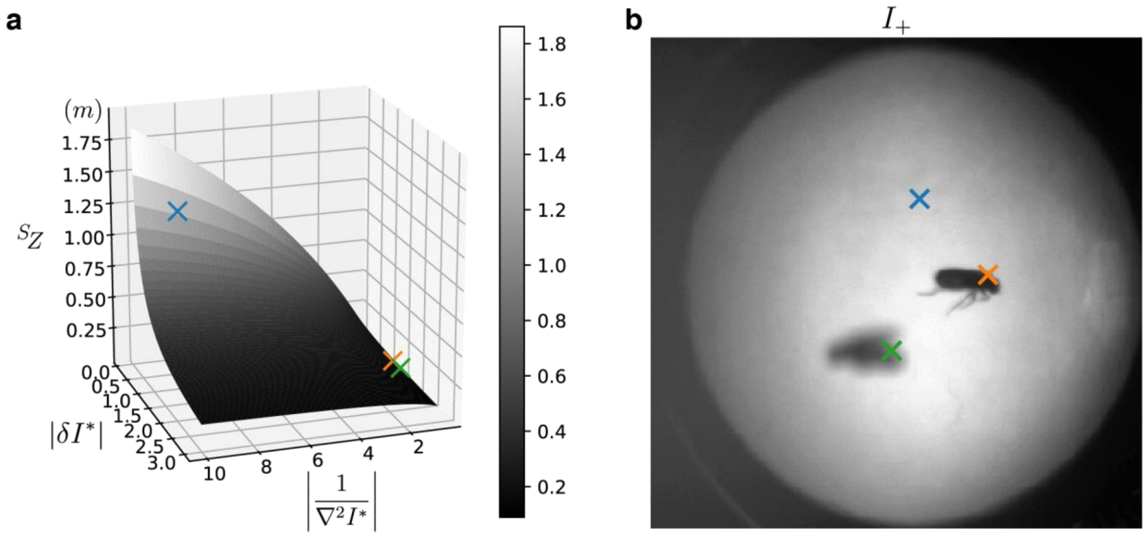

Fig. S3. Modeling depth error. (a) First-order approximation of the standard deviation of measured depth s_Z of the metalens sensor as a function of processed image values $(|1/\nabla^2 I^*|,\ |\delta I^*|)$, based on a simple additive Gaussian model for sensor noise. (b) A sample scene. The standard deviation of measured depth s_Z at any pixel can be estimated using equation (S17) and for three particular pixels, is visualized in (a). The blue pixel is in a low-contrast region where the standard deviation is large (and confidence is low). The orange and green pixels are in high-contrast regions where standard deviation is small (and thus confidence is high).

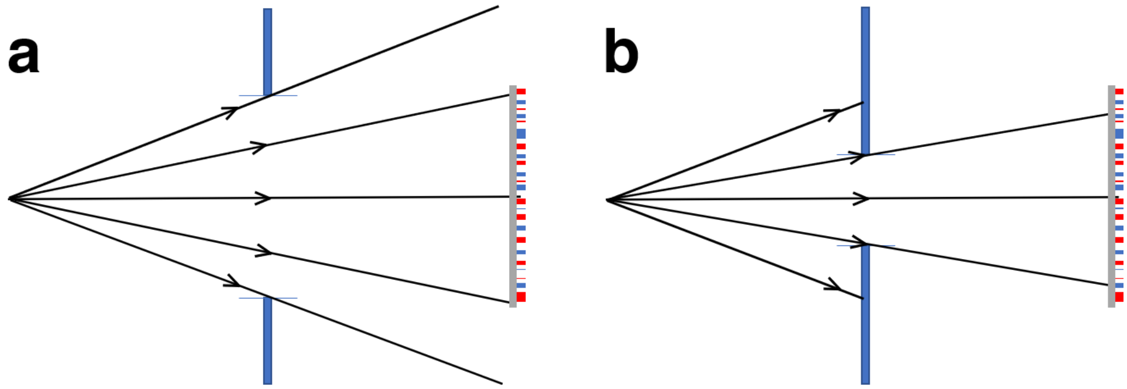

Fig. S4. Two possible configurations of the entrance pupil. (a) The metalens functions as the entrance pupil of the optical system. In this case, the on-axis angular acceptance range of the light cone is determined by the metalens, not the rectangular aperture. (b) The rectangular aperture functions as the entrance pupil of the optical system. In this case, the on-axis angular acceptance range of the light cone is limited by the rectangular aperture.

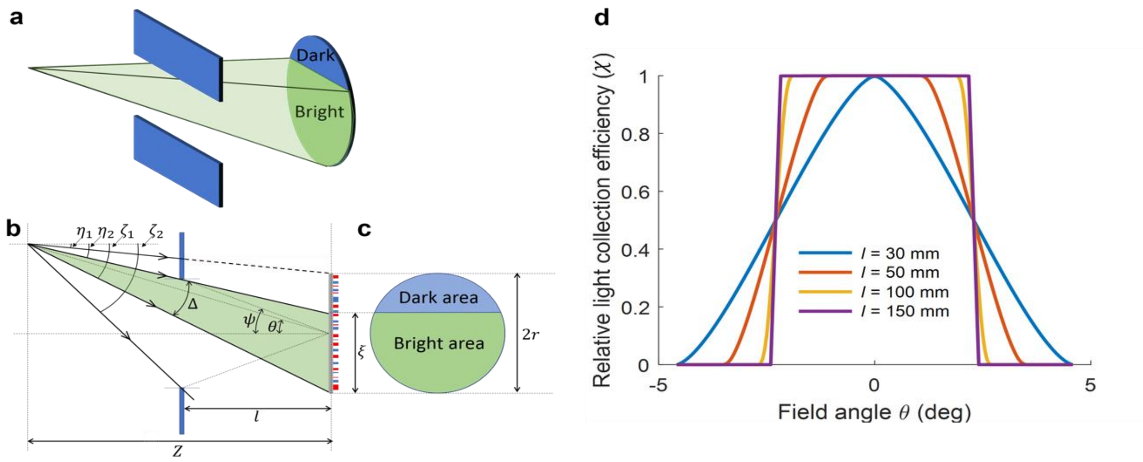

Fig. S5. Vignetting caused by rectangular aperture. (a) Part of the metalens is occluded by the rectangular aperture for off-axis incident light. (b) Side view of optics. The light collection efficiency is determined by four angles: η_1 and η_2 are, respectively, the tilt angles of the lines connecting the object point with the top edges of the metalens and the rectangular aperture; ζ_1 and ζ_2 are the tilt angles of the lines connecting the object point with the bottom edges of the metalens and the rectangular aperture. The field angle is θ, and ψ is the angular size of the rectangular aperture relative to the metalens center. The tangential angular Δ is the range of light that can be captured by the system. (c) Front view of the metalens. When light is blocked by the rectangular aperture, only part of the metalens is illuminated, which is denoted as the bright area. (d) The relative light collection efficiency as a function of field angle. Different lines correspond to different distance between the rectangular aperture and the metalens. For this graph, the angular size of the aperture is fixed at $\psi = 2.3°$, which is approximately the angular size of the aperture in our setup.

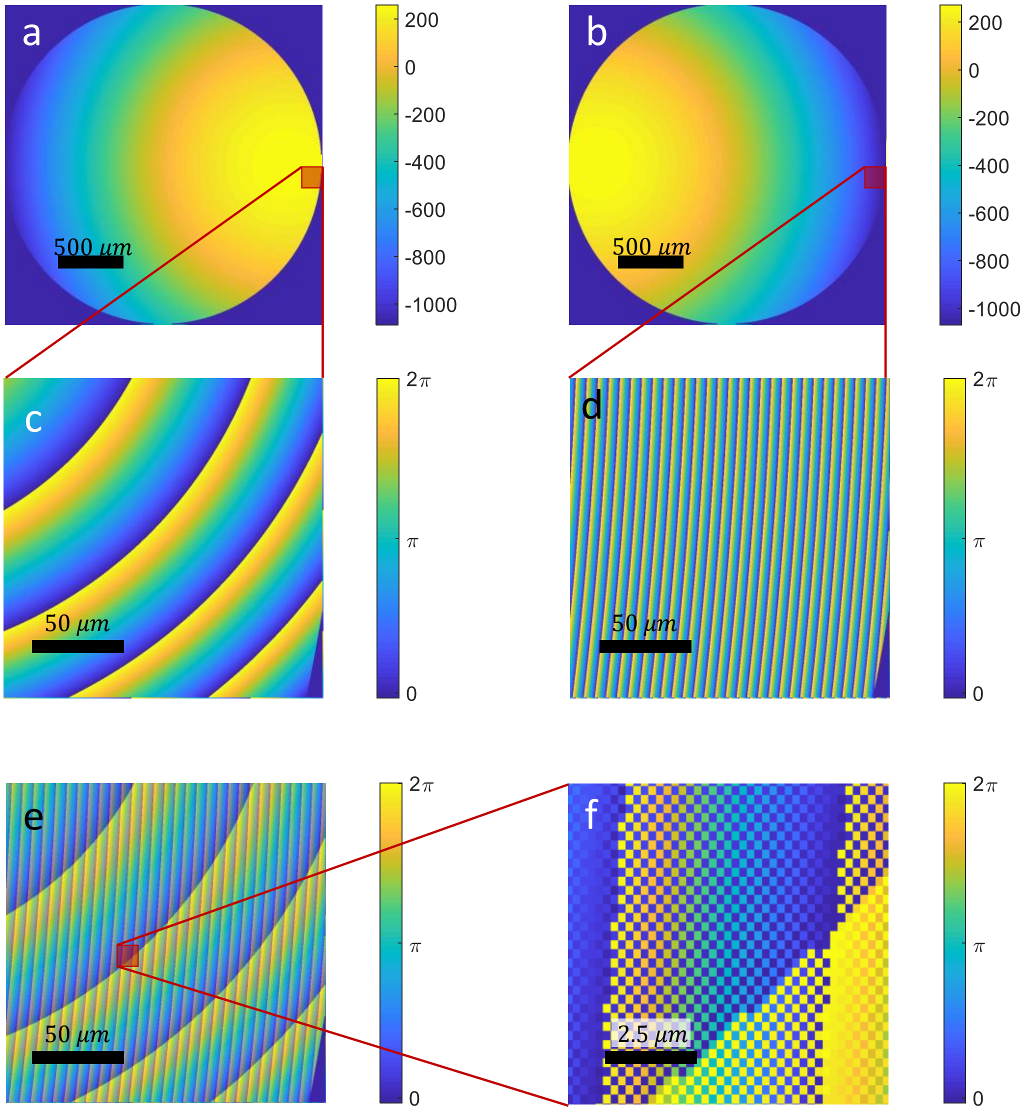

Fig. S6. The multiplexed, wrapped phase profile of the metalens. (a)-(b) The *unwrapped* phase for the two off-axis lens phase profiles $\phi_{\pm}$ respectively, following equation (S24). The final metalens phase profile is the spatial interleaving of the two phase profiles. (c)-(d) Zoom-in view of the *wrapped* phase for the two off-axis lens phase profiles in the same region on the metalens (the red highlighted area in (a)-(b)). Note the difference in the orientation and spacing of Fresnel zones. (e) The spatial interleaving result of (c) and (d). (f) Zoom-in view of the spatially interleaved wrapped phase profile in the highlighted red region in (e).

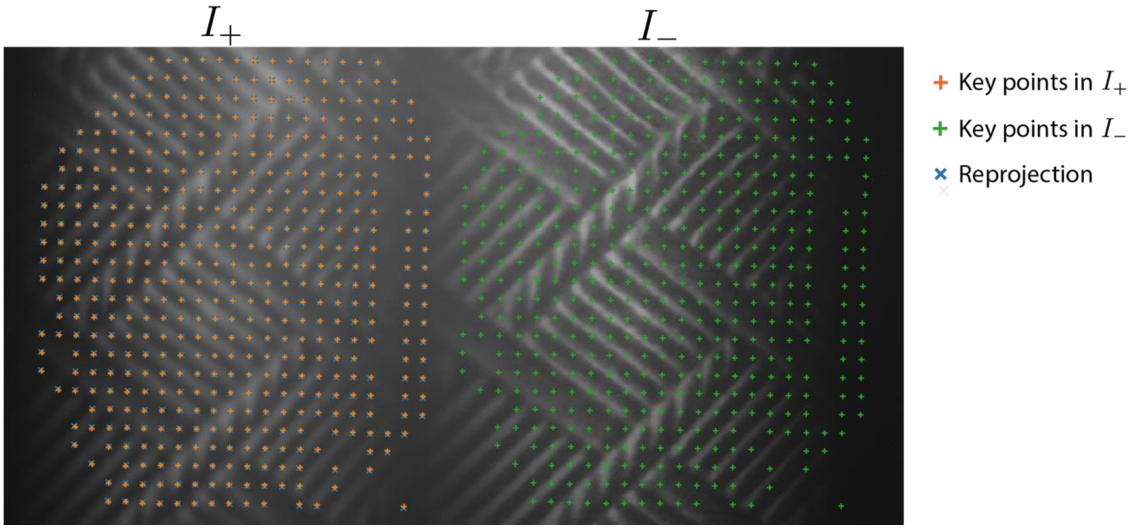

Fig. S7. Alignment of the image pair (I_+, I_-). We use a shifting point light source in front of the sensor to produce a dense set of corresponding points between the two adjacent regions of the photo sensor (orange and green), and we use these correspondences to fit a linear projective transformation (homography) that maps pixel locations in I_- to corresponding pixel locations in I_+. Experimentally we find that the fitted homography does not change when the light sources are placed at different depths. After fitting, the mean alignment error between I_+ and I_- (blue) is 0.8 pixels.

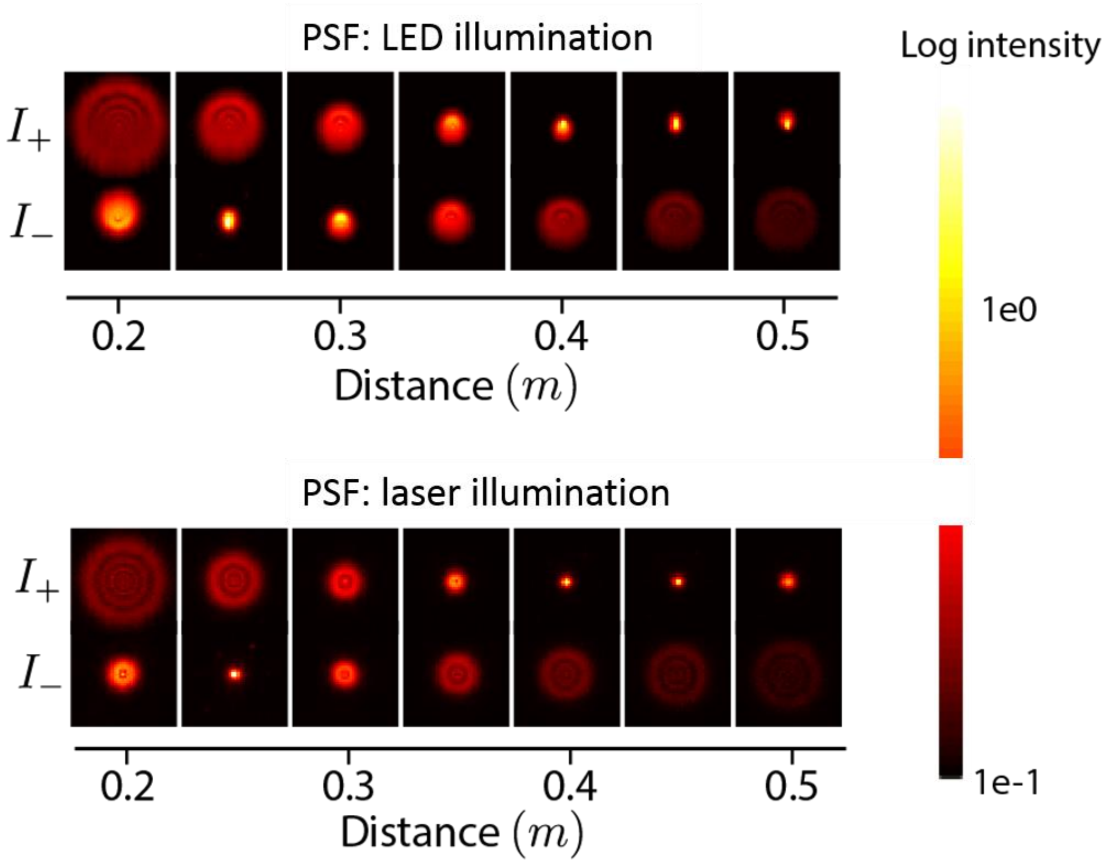

Fig. S8. Point spread functions of the sensor, measured using LED and laser sources. In general, the shapes of the PSFs resemble pillboxes, but with ringing. The PSFs from LED sources are subject to chromatic aberration and are therefore asymmetric.

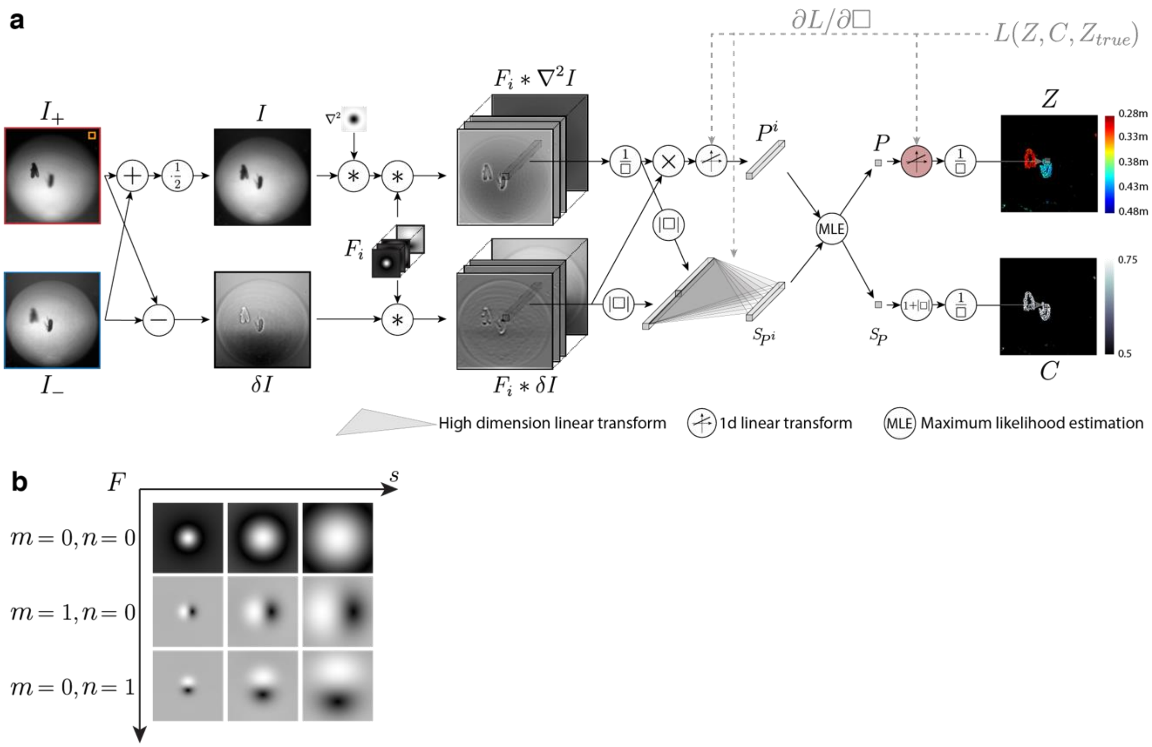

Fig. S9. Feedforward computational graph. (a) Each pair of (aligned) input images (I_+, I_-) is converted into a per-pixel depth map Z and a per-pixel confidence map C. In total, there are fewer than 700 floating point operations (FLOPs) required for each output pixel, and the spatial support ("receptive field") required to generate one pixel point of depth and confidence is 25×25 pixels (orange box in I_+). The feed-forward structure of the calculations allows the simultaneous tuning of all parameters by back-propagation and gradient descent. Most parameters are optimized using simulated data, and only two parameters (red) are fine-tuned using captured measurements. (b) Expanded view of the filter bank F.

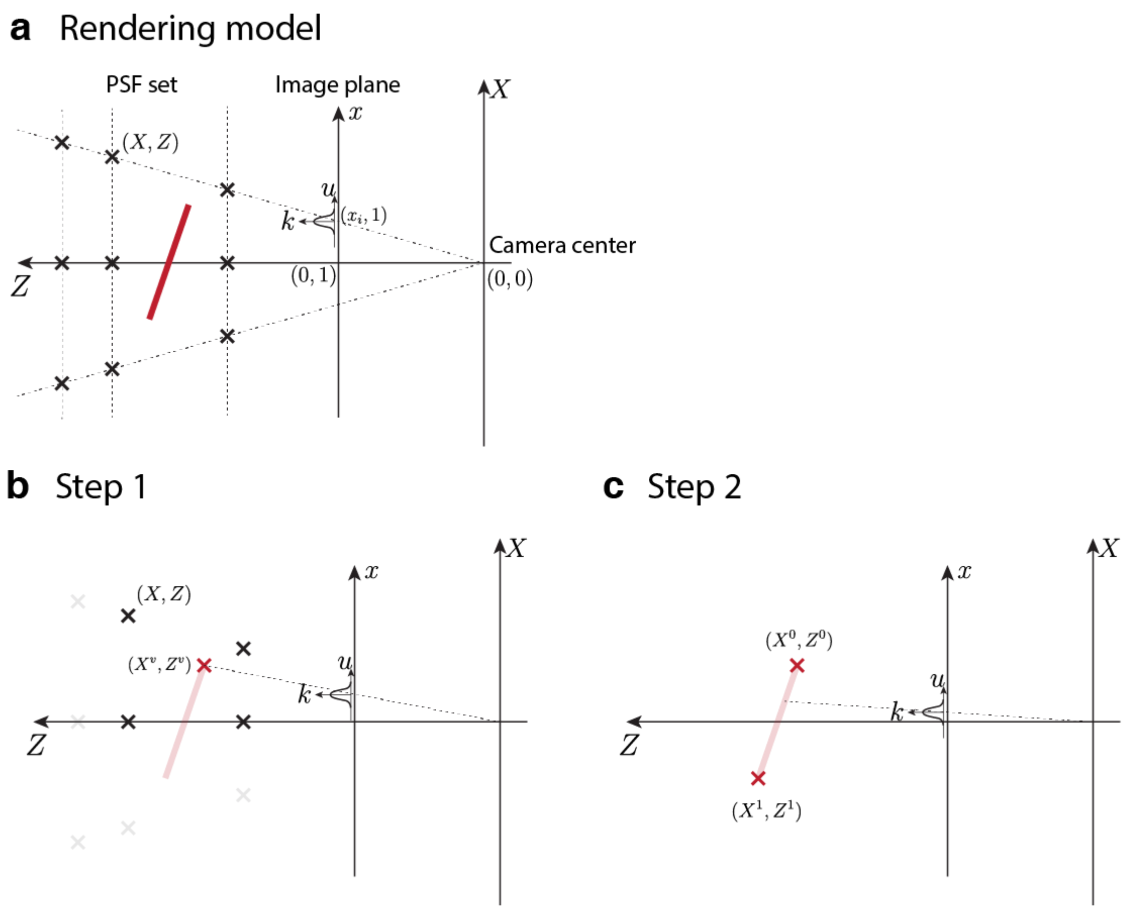

Fig. S10. Rendering defocused images of a single linear segment in two dimensions. (a) The renderer takes as input a set of tabulated PSFs corresponding to a discrete set of point source locations (X, Z). To generate an image of the line segment (red), the system first approximates the PSFs at the vertices (X^v, Z^v) by spatial interpolation (b). Then, it interpolates the PSF at every point in the line segment using the PSFs at the vertices, sum the contributions from each point weighted by the texture (emitted radiance) at that point (c).

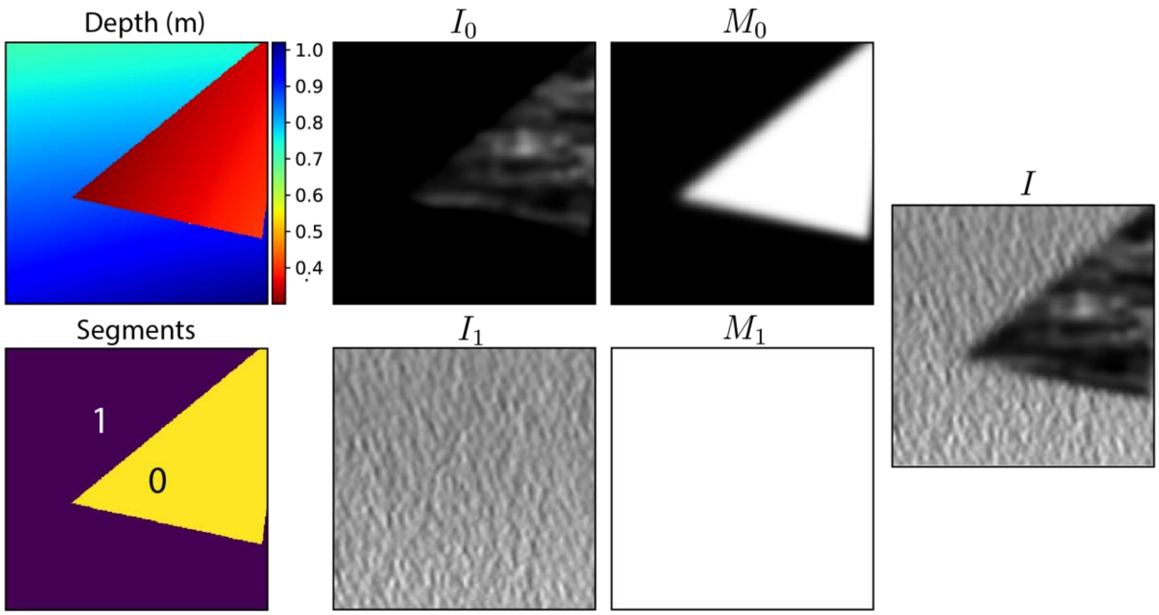

Fig. S11. Sample scene from our renderer. Our renderer generates image of foreground (I_0) and background (I_1) separately using equation (S38), and combine them together using the masks (M_0, M_1) according to equation (S39). This scheme closely approximates the scene at the depth boundaries.

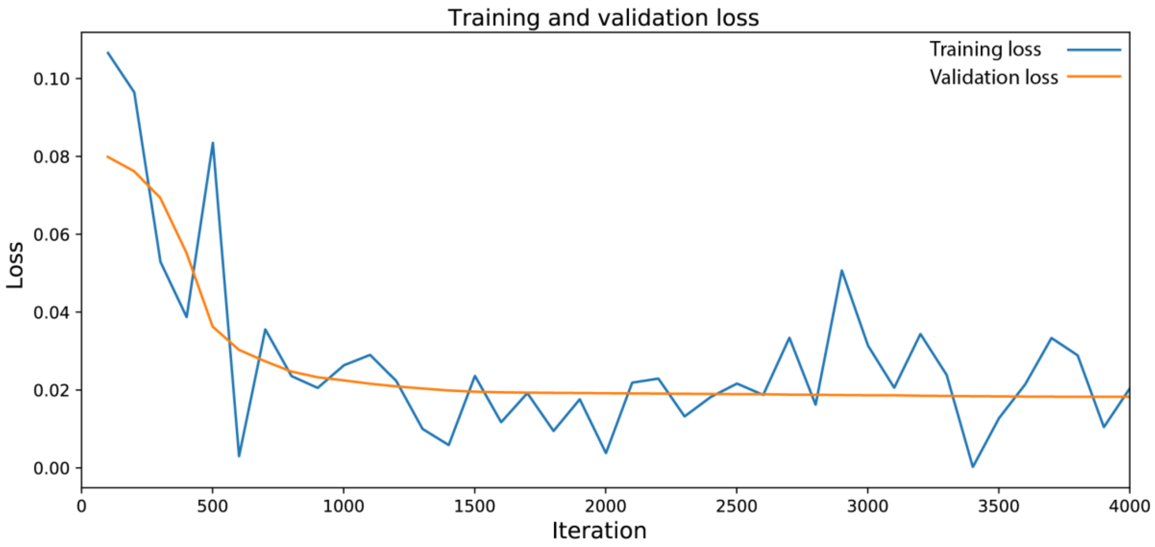

Fig. S12. The training and validation loss on the simulated dataset.

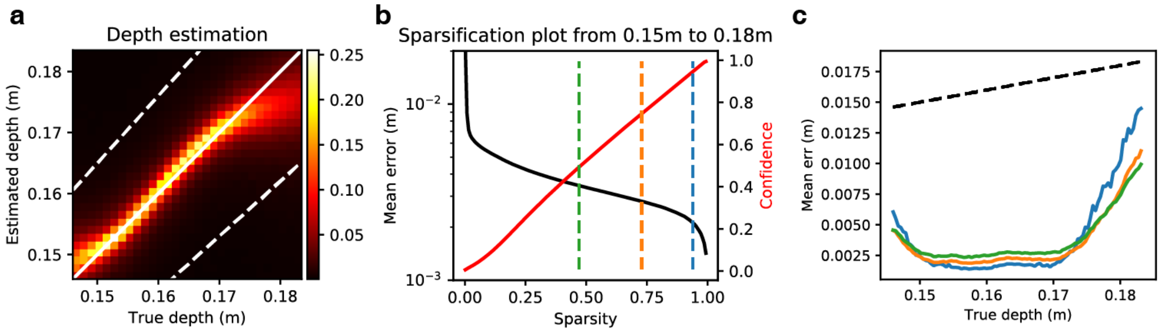

Fig. S13. Validation of the system in simulation. (a) The distribution of measured depth values for each true depth. Ideally all predictions should lie on the solid diagonal line. White dashed line indicate 10% relative error. (b) Sparsification plot created by sweeping a confidence threshold from 0 to 1 and only reporting depth at pixels with confidence above each threshold. For example, a confidence threshold (red curve) value of 0.75 corresponds to depth being reported at about 25% of pixels (75% sparsity on abscissa) and a mean error (black curve) value of about 0.002m. (c) Mean error as a function of object distance ("true depth"), plotted using three different confidence thresholds that are colored in correspondence with the dashed lines in (b). For object distances between 0.15m and 0.17m, where the system is most accurate, increasing the confidence threshold (sparsity) generally reduces the mean error.

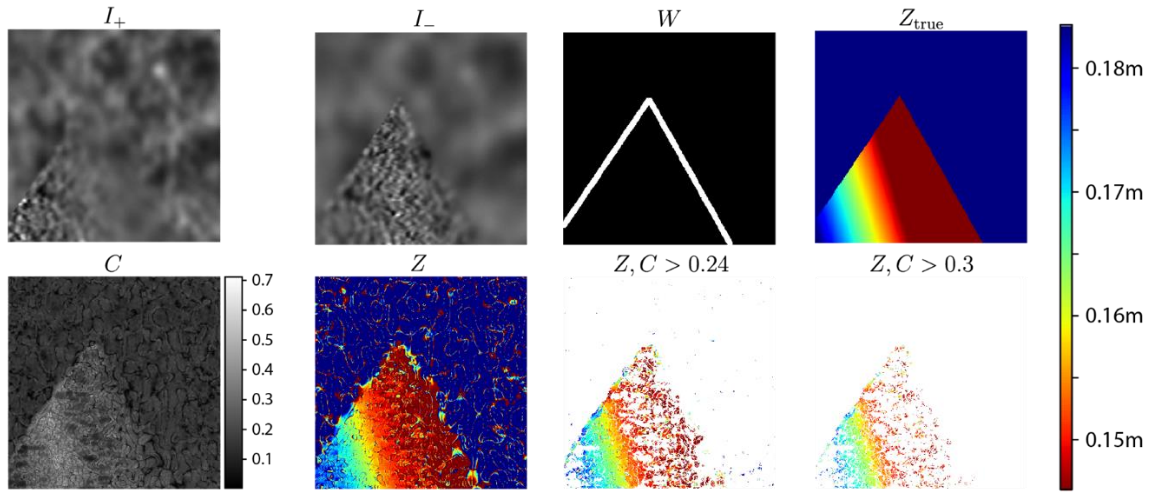

Fig. S14. Depth and confidence estimation of a sample simulated scene. The simulator rendered the image pair (I_+, I_-) given the true shape $\mathrm{Z}_{\mathrm{true}}$ and textures. The boundary mask W adds weight to the depth boundary in the loss function in equation (S42). We demonstrate the confidence mask C and the estimated depth Z under different confidence threshold/density.

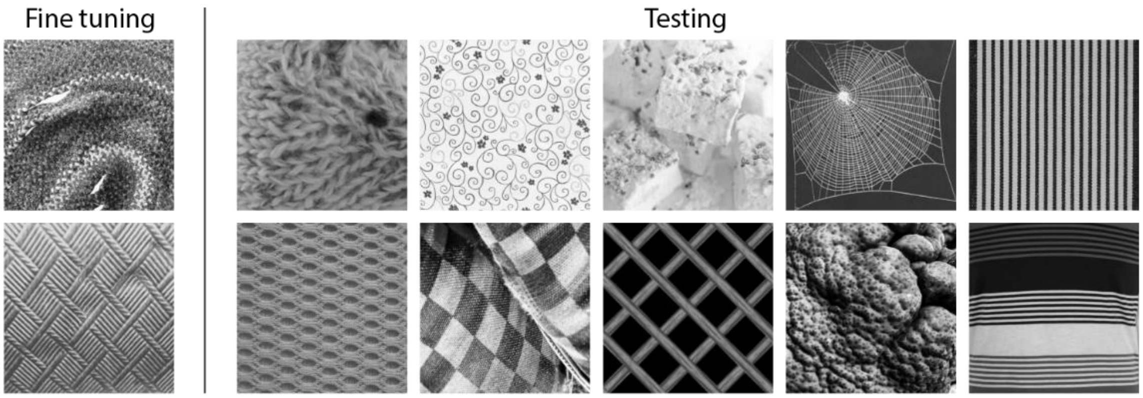

Fig. S15. Textures drawn from Describable Textures Dataset(6) used in fine tuning and testing of the real prototype.

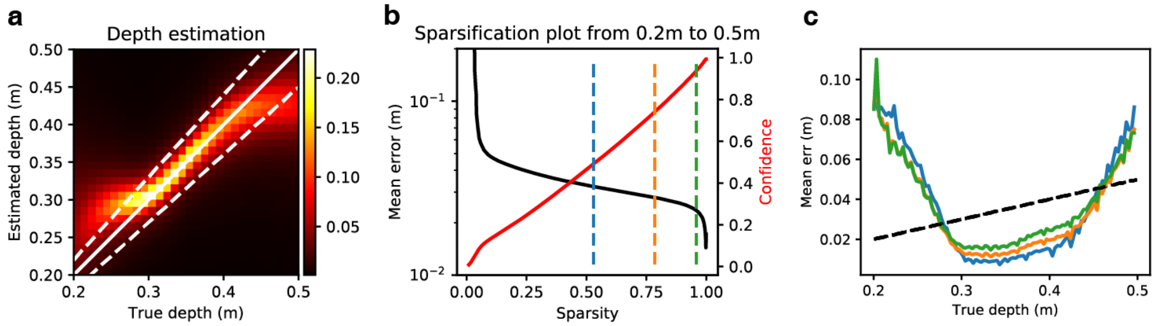

Fig. S16. Testing of the real prototype. Similar to Fig. S13, we provide quantitative evaluation of the depth accuracy of the real prototype, after fine tuning the parameters, using captured data with known depth. (a) The distribution of estimate depth at every true depth. (b) The sparsification plot. (c) Mean error at different confidence thresholds/sparsity indicated by the corresponding color in (b). According to (b) and (c), with the increase of confidence threshold/sparsity, the mean error reduces monotonically. Using the 50% most confidence pixels (green in (b) and (c)), the system measures depth throughout a range of 0.3m to 0.4m within 10% relative error.

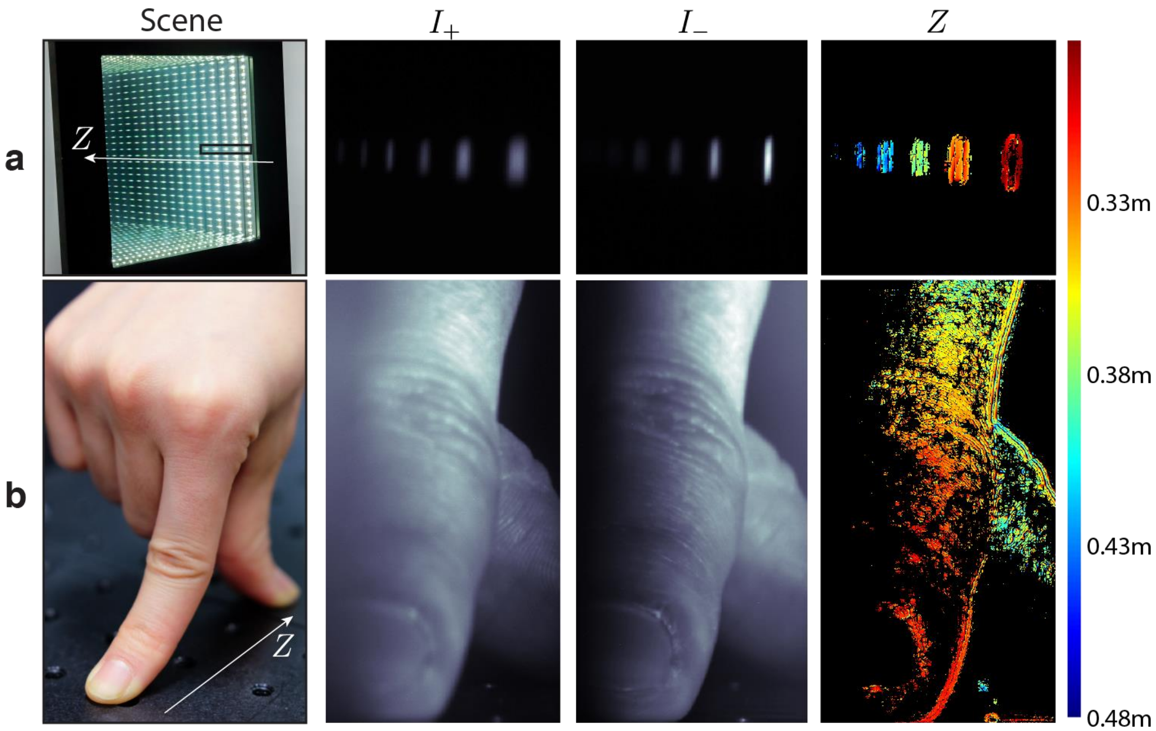

Fig. S17. Additional depth estimation results. (a) Reflective objects. The infinite mirror creates many virtual images of the light source at uniformly-stepped depth by reflecting the light back-and-forth inside the device. The depth sensor observes a part of it (black box), and measures the depth of the virtual images. (b) Finger gesture, which suggests use in a gesture-based interface for watches or other small, wearable devices.

Movie S1. The video shows real time depth estimation using the metalens depth sensor for several dynamic scenes. Similar to Fig. S17, it simultaneously shows the captured image pairs $(\boldsymbol{I}_+, \boldsymbol{I}_-)$, and the measured depth map $\boldsymbol{Z}$ masked using the confidence metric.

References

1. Guo Q, Alexander E, & Zickler T (2017) Focal Track: Depth and Accommodation with Oscillating Lens Deformation. *2017 IEEE International Conference on Computer Vision (ICCV)*, (IEEE), pp 966-974.
2. Alexander E (2018) A theory of depth from differential defocus. Doctor of Philosophy (Harvard University, Cambridge).
3. Burt P & Adelson E (1983) The Laplacian pyramid as a compact image code. *IEEE Transactions on Communications* 31(4):532-540.
4. Srinivasan PP, Garg R, Wadhwa N, Ren N, & Barron JT (2017) Aperture supervision for monocular depth estimation.
5. Dana KJ, Van Ginneken B, Nayar SK, & Koenderink JJ (1999) Reflectance and texture of real-world surfaces. *ACM Transactions On Graphics (TOG)* 18(1):1-34.
6. Cimpoi M, Maji S, Kokkinos I, Mohamed S, & Vedaldi A (2014) Describing textures in the wild. *Proceedings of the IEEE Conference on Computer Vision and Pattern Recognition*, pp 3606-3613.
